# Differential H3K4me3 Domains in Normal and Colorectal Cancer Cells Reveal Novel Epigenetic Targets

**DOI:** 10.3390/ijms26062546

**Published:** 2025-03-12

**Authors:** Ravinder Kaur Bahia, Camila Lopez, Gino Nardocci, James R. Davie

**Affiliations:** 1 Arnie Charbonneau Cancer Institute, Cumming School of Medicine, University of Calgary, Calgary, AB T2N 4N1, Canada; ravinder.bahia@ucalgary.ca; 2 Department of Cell Biology and Anatomy, University of Calgary, Calgary, AB T2N 4N1, Canada; 3 Department of Biochemistry and Medical Genetics, Rady Faculty of Health Sciences, Max Rady College of Medicine, University of Manitoba, Winnipeg, MB R3E 0J9, Canada; camila.lopezmoreno@gmail.com; 4 School of Medicine, Faculty of Medicine, Universidad de los Andes, Santiago 7620001, Chile; 5 Molecular Biology and Bioinformatics Lab, Program in Molecular Biology and Bioinformatics, Center for Biomedical Research and Innovation (CIIB), Universidad de los Andes, Santiago 7620001, Chile; 6 IMPACT, Center of Interventional Medicine for Precision and Advanced Cellular Therapy, Santiago 7620001, Chile; 7 Paul Albrechtsen Research Institute, CancerCare Manitoba, Winnipeg, MB R3E 0V9, Canada

**Keywords:** histone H3 trimethylated at lysine 4 (H3K4me3), colon cancer, cell adhesion, nervous system development, long non-coding RNA

## Abstract

Histone H3 trimethylated at lysine 4 (H3K4me3) is an histone mark associated with transcriptionally active genes. H3K4me3 has two types of distribution: a sharp distribution of approximately 500 bp and a broad H3K4me3 domain that may extend 4 kb and longer through the gene body. Most transcribed genes have a narrow H3K4me3 configuration, whereas genes involved in cell identity and tumor suppression have a broad arrangement in normal cells. In cancer cells, genes that promote cancer possess a broad H3K4me3 domain. In this study, we performed H3K4me3 chromatin immunoprecipitation sequencing to determine the genes with narrow and broad H3K4me3 configurations in normal colon epithelial cells and three colon cancer cell lines. The analysis revealed that genes involved in cell adhesion and nervous system development had an H3K4me3 peak next to their transcription start site in normal cells but not in colon cancer cells. Genes coding for long non-coding RNA (lncRNA) were differentially marked with a broad H3K4me3 domain in normal colon versus colon cancer cells (*FENDRR* in normal colon; *ELFN1-AS1* in colon cancer). Identifying the genes that are silenced or activated, particularly in colon cancer, provides a list of actionable targets for designing effective treatments for this prevalent human disease.

## 1. Introduction

Chromatin structure and function are modified by epigenetic processes, including histone post-translation modifications (PTMs), DNA modifications, chromatin-modifying complexes, and transcription factors. These processes determine which genes are transcribed. One notable histone PTM associated with transcribed genes is histone H3 trimethylated at lysine 4 (H3K4me3). Nucleosomes with H3K4me3 are typically found in a narrow (also called sharp) peak immediately downstream of the transcription start site (TSS) [1]. The chromatin-modifying enzymes, lysine methyltransferase KMT2A, KMT2F, and KMT2G, are among the enzymes that catalyze the formation of H3K4me3. These KMT complexes have modules or proteins that bind to unmethylated CpG islands, which are often present at the TSS. H3K4me3 can be demethylated by a family of lysine demethylases (KDM5); however, the demethylation rate is very low [2,3]. The balance of KMTs trimethylating H3K4 and KDM5 family members is often altered in cancer cells, resulting in changes in the steady state levels of H3K4me3 [4,5]. The H3K4me3 is recognized by several proteins called readers [6]. H3K4me3 readers are often in multiprotein complexes such as the MOZ/MORF lysine acetyltransferase (KAT6) complex, which acetylates H3K23, the SAGA KAT complex, which acetylates H3K9 and K14, and the histone deacetylase (HDAC) complex Sin3. The recruitment of KATs (KAT2A, 4, 5, 6, 7, 8, and 13B) and HDACs produces a dynamically acetylated H3K4me3 nucleosome [7] and a new set of chromatin readers (e.g., INTS11). Recruitment of chromatin remodelers, such as CHD1 and CHD6, to the H3K4me3-modified nucleosome further adds to the structural dynamics of this modified nucleosome. The H3K4me3 mark also repels repressive complexes, including PRC2 (producing the repressive mark H3K27me3) and the HDAC complex NuRD. The H3K4me3-modified nucleosome plays a role in transcription initiation, RNA polymerase II pause release, and elongation [4].

Recent studies suggest that the H3K4me3 PTM may also present as a lengthy continuum of H3K4me3-modified nucleosomes, which covers an extensive part of the coding region of the expressed gene [8]. This pattern of H3K4me3-modified nucleosomes is referred to as the broad H3K4me3 domain. Genes with the broad H3K4me3 domain are involved in cell identity in normal cells. In contrast, genes marked this way in cancer cells are involved in oncogenesis [8,9,10].

Colorectal cancer is the fourth most commonly diagnosed cancer in Canada, the second leading cause of cancer-related death in men and the third leading cause of cancer death in women. The increasing rates of colorectal cancer in young adults are concerning. *KRAS* and *BRAF* mutations are often present in colorectal cancer; the oncoprotein products of these genes result in increased activity of RAS-mitogen-activated protein kinase. Most colorectal cancers have chromosomal instability (CIN), whereas a smaller percentage of colorectal cancers exhibit the microsatellite instability (MSI) phenotype [11]. Recent studies have identified an association between H3K4me3 broad domains and chromatin accessibility of genes involved in oncogenesis [8,9,10]. However, the distribution of H3K4me3 broad domains over the potential gene targets involved in the development and progression of colorectal cancers is poorly understood.

In this study, we profiled H3K4me3-modified nucleosomes in three colorectal cancer cell lines, normal colonic epithelial cell lines, and normal colon tissue to identify differentially marked genes with a narrow or broad H3K4me3 pattern.

## 2. Results

### 2.1. H3K4me3 Distribution in Normal Colon Versus Colorectal Cancer Cells

**Figure 1:**
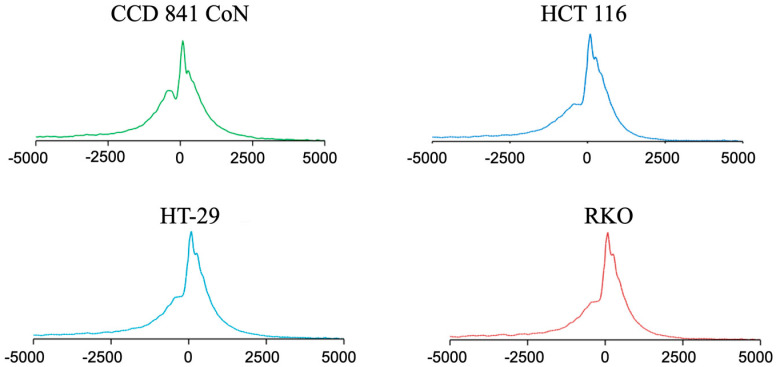
The profile of H3K4me3 peaks around the transcription start sites (TSS) in the CCD 841 CoN (normal colon) and colorectal cancer cells (HCT116, HT-29, RKO). Histone mark enrichment distribution near the TSS (±5 kb).

**Figure 2:**
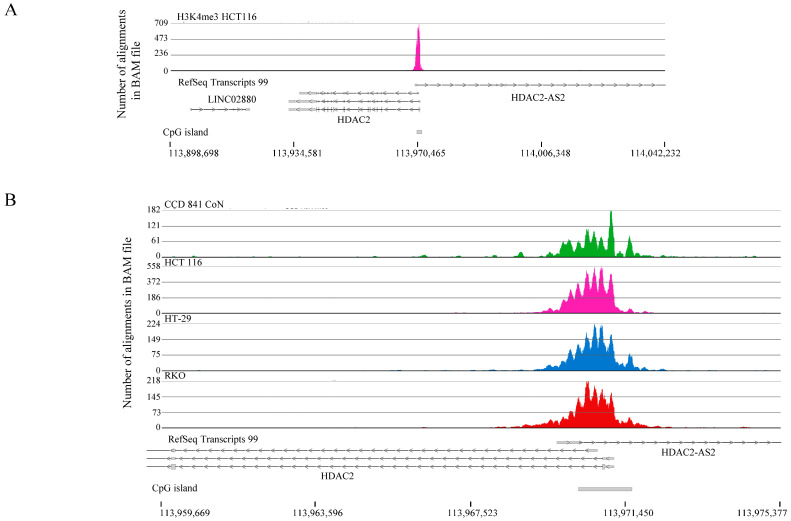
H3K4me3 chromatin immunoprecipitation sequencing tracks for *HDAC2* gene in CCD 841 CoN (normal colon) and colorectal cancer cells (HCT116, HT-29, RKO). The position of CpG islands is indicated. (**A**) *HDAC2* gene, (**B**) *HDAC2* gene, zoom in view.

**Figure 3:**
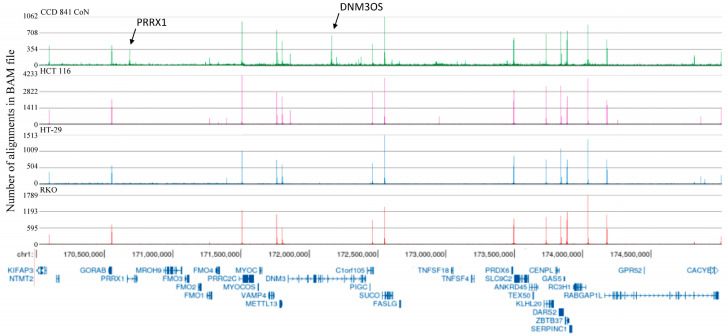
H3K4me3 chromatin immunoprecipitation sequencing tracks for chromosome 1:170,500,000 to 174,500,000 in CCD 841 CoN (normal colon) and colorectal cancer cells (HCT116, HT-29, RKO). Genes with H3K4me3 peaks only in normal CCD 841 cells are indicated with an arrow.

To identify the H3K4me3 differentially marked genes, we first assessed the levels of H3K4me3 in normal colon epithelial versus colon cancer cells. We showed no apparent difference in the levels of H3K4me3 in CCD 841 CoN, HCT 116 cells, HT-29 cells, and RKO cells (Supplementary Figure S1). ChIP sequencing was performed using the input and anti-H3K4me3 antibody-immunoprecipitated mononucleosomes from these four cell lines (Supplementary Figures S2–S5). The Partek Flow (version 12.4.3) pipeline (MACS3 default setting) produced the following peaks: CCD 841 CoN (42,853), HCT 116 cells (43,090), HT-29 cells (46,902), and RKO cells (98,837). The distribution of the peaks relative to the TSS was similar for the four cell lines (Figure 1). The most intense H3K4me3 peaks were located after TSS. In normal and colorectal cancer cell lines, the histone deacetylase 2 (*HDAC2*) gene had a narrow H3K4me3 peak after the first exon (Figure 2A). Upon closer examination, it is evident that the narrow peak consists of seven to eight well-positioned H3K4me3-modified nucleosomes (Figure 2B). Figure 3 takes a broader view, showing a 5 million bp region of chromosome 1. The distribution of H3K4me3 peaks over this genomic region was similar for the four cell lines; however, H3K4me3 peaks were often observed in the normal CCD 841 CoN that were absent in the colorectal cancer cell lines (see arrows in Figure 3), suggesting a differential gene regulation in the colorectal cancer cell lines compared to normal colon cells.

### 2.2. H3K4me3-Marked Genes Associated with Cell Adhesion and Nervous System Development Were Unique in Normal Colon Cells

To further expand the dataset for H3K4me3 genomic locations in the normal colon, FASTQ files from H3K4me3 ChIP sequencing studies of human sigmoid and transverse colon tissues were put through the Partek Flow pipeline. The genomic distribution of H3K4me3 peaks was similar among normal colon tissues and the CCD 841 CoN cell line (Supplementary Figure S6). Of the four normal colon tissues, H3K4me3 ChIP seq for the sigmoid colon (sigmoid colon H3K4me3_2) had the best signal-to-noise ratio and was used in subsequent analyses.

**Figure 4:**
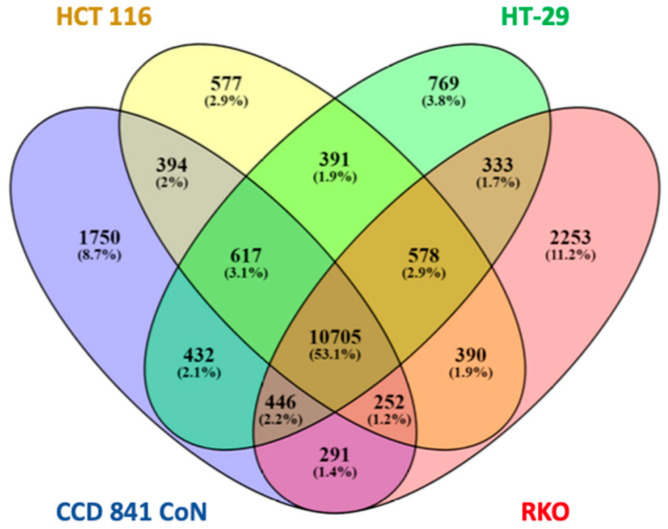
The distribution of genes with an H3K4me3 peak includes the TSS in CCD 841 CoN (normal colon) and colorectal cancer cells (HCT116, HT-29, RKO).

**Figure 5:**
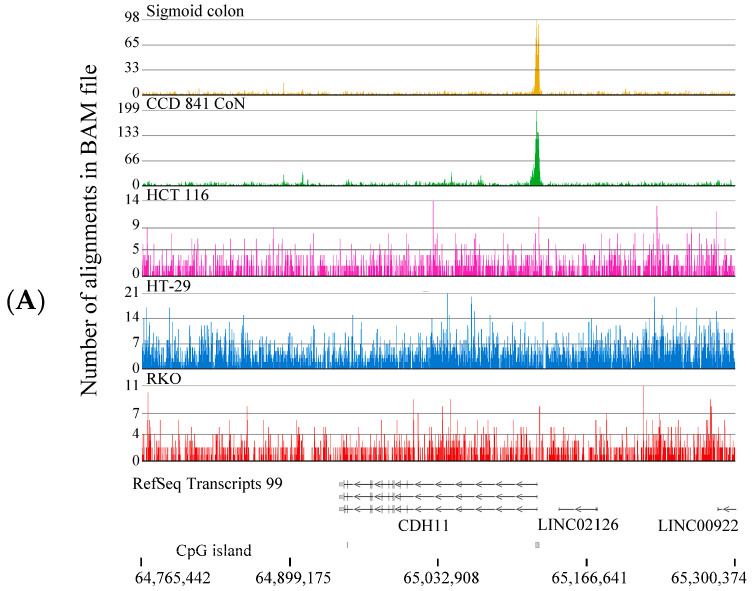

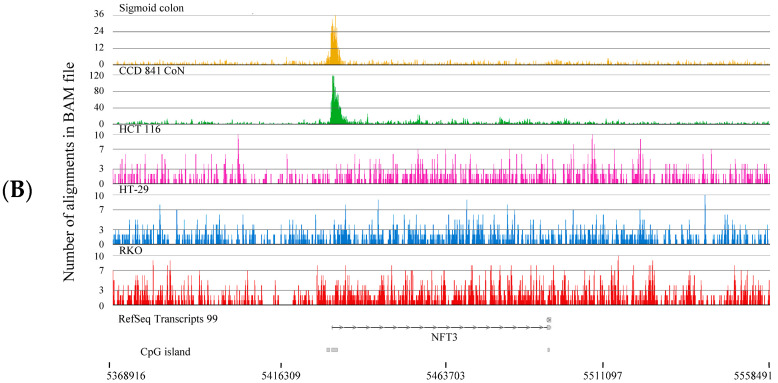
H3K4me3 ChIP-Seq tracks of genes in CCD 841 CoN (normal colon) and colorectal cancer cells (HCT116, HT-29, RKO). (**A**) *CDH1*, (**B**) *NTF3*. Positions of CpG islands are indicated.

To identify the genes with H3K4me3 peaks in the normal colon epithelial cells absent in the colon cancer cell lines, we restricted the analyses to genes with H3K4me3 peaks next to the TSS. The gene IDs for the four cell lines were entered into the Venny 2.1.0 program (Figure 4). This analysis identified 1750 genes with a TSS-associated H3K4me3 peak in CCD 841 CoN cells, but not in colorectal cancer cell lines. This list of genes was cross-referenced to genes with H3K4me3 peaks in normal sigmoid colon tissue to identify common genes. This analysis yielded 1115 genes. Gene ontology (biological process) analysis of this set of genes using David and GeneCodis identified cell adhesion and nervous system development as the most significant processes (Table 1). Genes involved in cell adhesion included the MAM domain-containing glycosylphosphatidylinositol anchor 2 gene, *MDGA2*, and cadherin genes *CDH2*, 6, 11, and 13 (Figure 5A shows *CDH11*). Genes involved in nervous cell development included Rho GTPase-activating protein 26 (*ARHGAP26*; also named *GRAF1*) and neurotrophin 3 (NTF3) (Figure 5B). The *CHDH11* and *NTF3* genes had H3K4me3 peaks at the 5′ end, overlapping with a CpG island. These genes did not have H3K4me3 peaks in colorectal cancer cells (Supplementary Table S3).

**Figure 6:**
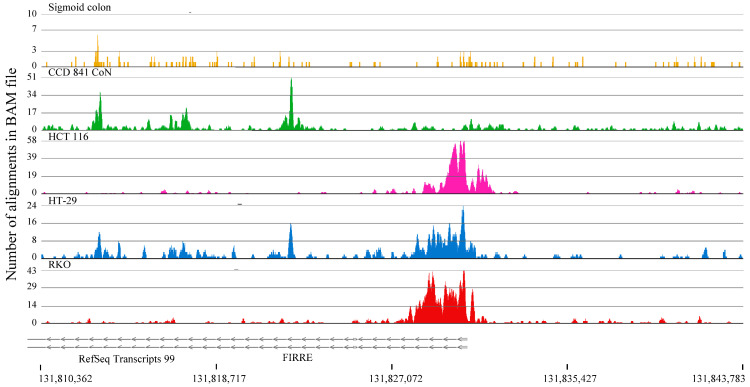
H3K4me3 ChIP-Seq track for the *FIRRE* gene in CCD 841 CoN (normal colon) and colorectal cancer cells (HCT116, HT-29, RKO).

The same strategy was applied to identify genes with H3K4me3 peaks in the three colorectal cancer cell lines but not in normal colon cells. Upon inspecting the tracks for these genes, we observed many false positives (i.e., the gene of interest also had H3K4me3 peaks in normal colon samples). However, we did identify the FIRRE intergenic repeating RNA element (*FIRRE*) gene as having H3K4me3 peaks in the three colorectal cancer cell lines but not in the normal colon samples (Figure 6), suggesting its differential gene activation and expression particularly in colorectal cancer cells. An elevated level of *FIRRE* has been reported to function as an oncogenic factor in colorectal cancer cells [12].

### 2.3. Broad H3K4me3 Domains in Normal Colon Versus Colorectal Cancer Cells

**Figure 7:**
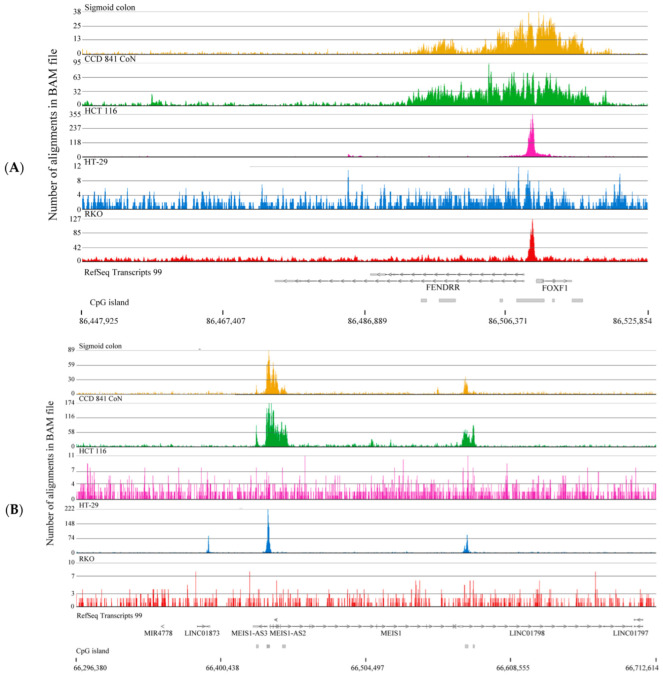
H3K4me3 ChIP-Seq tracks of genes in CCD 841 CoN (normal colon) and colorectal cancer cells (HCT 116, HT-29, RKO). (**A**) *FENDRR* and (**B**) *MEIS1*. Positions of CpG islands are indicated.

These analyses used the broad setting of the MACS3 peak finder. We attempted various approaches to identify genes with a broad H3K4me3 domain exclusive to normal colon or colorectal cancer cells. One approach used the MACS3 broad setting with input and ChIP-Seq files for each entered cell line. (We did not do this for the Sigmoid colon sample as an input was not provided.) Following annotation using Ensembl (version 91), the following H3K4me3 peaks were identified for each cell line: CCD 841 CoN, 47830; HCT 116, 33740; HT-29, 35326; and RKO, 54804. The range of H3K4me3 peak lengths was CCD 841 CoN, 146 to 24,966 bp; HCT 116, 159 to 65,678; HT-29, 158 to 85,834; RKO, 167 to 669,379. Genes with the top 5% broadest H3K4me3 domains that included the TSS were identified (CCD 841 CoN, 1002; HCT116, 888; HT-29, 898; RKO, 1046). David and GeneCodis’ analyses identified the key biological functions for the top 5% of genes for each cell line as follows: CCD 841 CoN (regulation of transcription), HCT 116 (regulation of transcription, regulation of cell cycle), HT-29 (regulation of transcription), and RKO (protein localization of plasma membrane, chromatin remodeling, protein phosphorylation). Genes with a broad H3K4me3 domain unique to CCD 841 CoN (731), HCT 116 (318), HT-29 (266), and RKO (563) were identified using VENNY 2.1 (Supplementary Figure S7). The H3K4me3 tracks for each group of genes were visualized using the Partek Flow chromosome view to confirm that the gene with the broad H3K4me domain was unique to the cell line. Genes with a broad H3K4me3 domain in normal CCD 841 CoN cells but not in colorectal cancer cells included *FOXF1* adjacent non-coding developmental regulatory RNA (*FENDRR*) and Meis Homeobox 1 (*MEIS1*) (Figure 7A,B, respectively; Supplementary Figure S8). Other genes with a broad H3K4me3 domain only in the CCD 841 CoN included *FILIP1L*, *ZEB2*, *NKX2-3*, *NR3C1*, *F3*, *COL1A2*, *DCN*, *TBX2*, and *LBH* (Supplementary Table S4). These genes also have a broad H3K4me3 domain in the normal sigmoid colon. This approach effectively identified genes with broad H3K4me3 domains only in the normal colon and not in colorectal cancer cells; however, several genes on the list were false positives. In contrast, this approach did not identify genes with broad H3K4me3 domains in the colorectal cancer cells and not normal colon cells (Supplementary Table S4).

**Figure 8:**
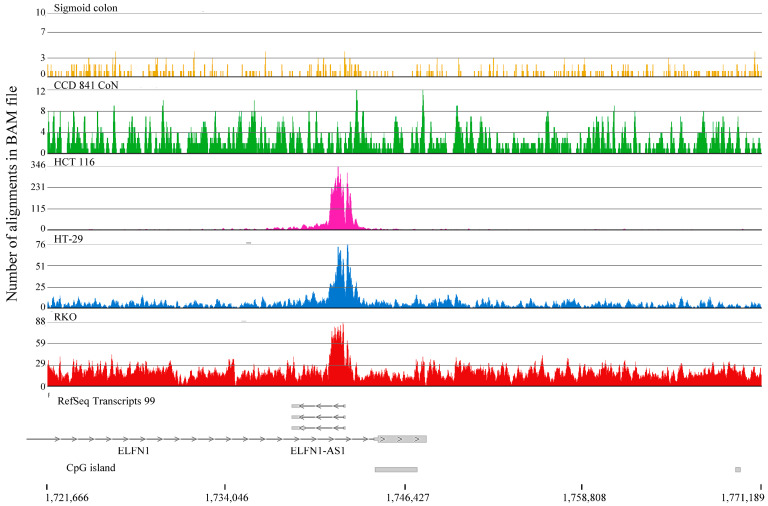
H3K4me3 ChIP-Seq track for the *ELFN1* gene in CCD 841 CoN (normal colon) and colorectal cancer cells (HCT116, HT-29, RKO). Positions of CpG islands are indicated.

The second approach was using MACS3 broad setting and entering the CCD 841 CoN, HCT 116, HT-29, RKO, and Sigmoid colon H3K4me3 aligned reads as the ChIP without input. Following annotation using Ensembl (version 91), the following H3K4me3 peaks were identified for each cell line: sigmoid colon, 33608; CCD 841 CoN, 33090; HCT 116, 18446; HT-29, 19843; RKO, 15371. The H3K4me3 peak lengths were as follows: sigmoid colon, 171–3617; CCD 841 CoN, 146–4949 bp; HCT 116, 159–4488; HT-29, 177–5070; RKO, 192–4234. The genes with H3K4me3 peaks were entered into Venny 2.1 to identify genes unique to the sigmoid colon or common to the colorectal cancer cell lines but absent in the normal colon (Supplementary Figure S9). This analysis identified 3795 genes unique to the sigmoid colon and 34 genes common to colorectal cancer cells, but absent in the normal colon. The track for each gene was visualized using Partek Flow’s chromosome view to validate the gene assignment. This approach successfully identified genes with an H3K4me3 peak in normal cells, but not in colorectal cancer cells [Teashirt Zinc Finger Homeobox 3 (*TSHZ3*) (Supplementary Figure S10), Adhesion G Protein-Coupled Receptor L3 (*ADGRL3*), Neural Cell Adhesion Molecule 1 (*NCAM1*), Erythrocyte Membrane Protein Band 4.1 Like 3 (*EPB41L3*), and OCA2 Melanosomal Transmembrane Protein (*OCA2*)] (Supplementary Table S5). Identifying genes with H3K4me3 peaks in colorectal cancer cell lines but not in normal colon tissue and the CCD 841 CoN cell line was more challenging. Upon inspecting the tracks, we found that many unique genes were false positives, and the gene had an H3K4me3 peak in all cells and tissues. However, we found a few genes with an H3K4me3 peak in the three colorectal cancer cell lines and not in the normal colon cells. The Solute Carrier Family 5 member 4 SLC5A4 Antisense RNA 1 (*SLC5A4-AS1*), Solute Carrier Organic Anion Transporter Family Member 1B3 (*SLCO1B3*), and ELFN1 Antisense RNA 1 (*ELFN1-AS1*) were identified as having an H3K4me3 peak in colorectal cancer cells but not in normal colon samples (Figure 8). These genes have been shown to promote oncogenesis in colorectal cancer through different mechanisms [13,14].

**Figure 9:**
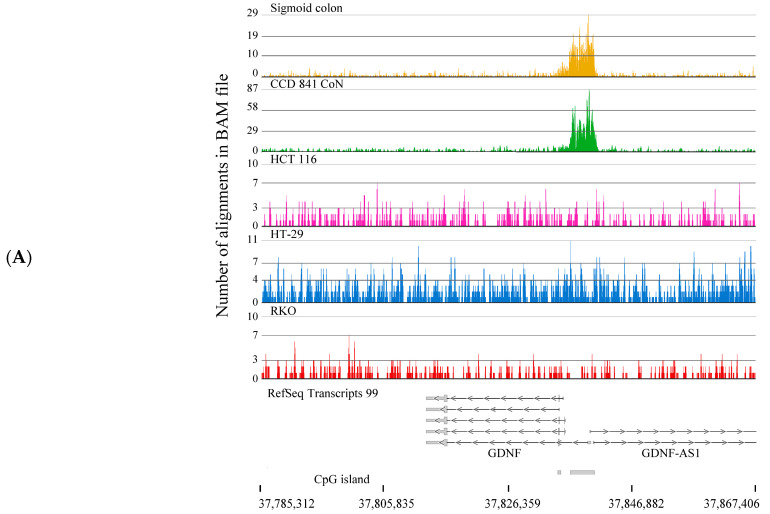

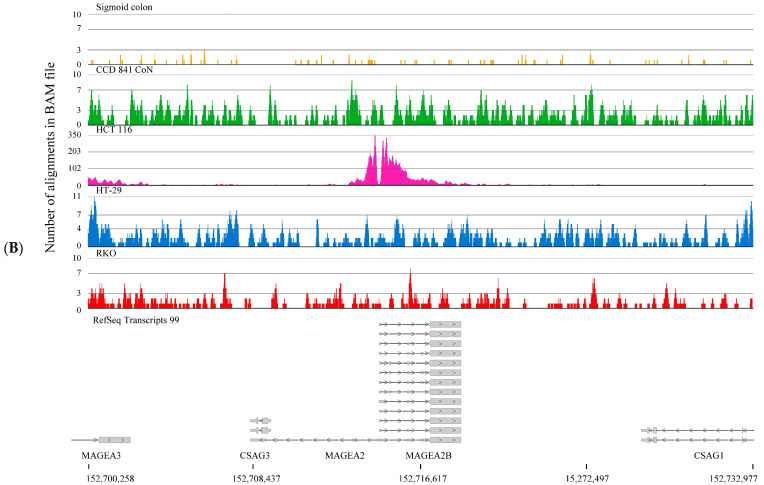
H3K4me3 ChIP-Seq tracks. H3K4me3 ChIP-Seq track for (**A**) *GDNF* and (**B**) *MAGEA2* genes in CCD 841 CoN (normal colon) and colorectal cancer cells (HCT116, HT-29, RKO). Positions of CpG islands are indicated.

Genes with the broadest H3K4me3 domains (top 5%) with TSS in the sigmoid colon (1126), CCD 841 CoN (864), HCT 116 (706), HT-29 (687), and RKO (611) were identified. We entered the gene sets for CCD 841 CoN, HCT 116, HT-29, and RKO into Venny 2.1 to identify genes unique to the cell line (Supplementary Figure S11) (CCD 841 CoN (447), HCT 116 (283), HT-29 (184), and RKO (173). Furthermore, this analysis produced a list of potential genes (79) unique to the colorectal cancer cells. Using Partek Flow chromosome viewer, we determined the validity of each gene in each group (Supplementary Table S5). This analytical pipeline effectively identified genes with a broad H3K4me3 domain in normal (sigmoid colon and CCD 841 CoN) but not in colorectal cancer cells. These included Glial Cell Derived Neurotrophic Factor (*GDNF*) (Figure 9A), protocadherin 18 (*PCDH18*), NALCN Channel Auxiliary Factor 1 (*NALF1*, also named *FAM155A*), DAM Metallopeptidase with Thrombospondin Type 1 motif 5 (*ADAMTS5*), and calponin 3 (*CNN3*). We also identified genes with a broad H3K4me3 domain in a specific colorectal cancer cell line but not in normal cells, which could be attributed to intertumoral heterogeneity or a cluster of genes on chromosome X in HCT 116 cells, e.g., MAGE Family Member A2 (MAGEA2) (Figure 9B). MAGEA2 was present in a cluster of genes (*GABRQ*, *MAGEA3*, *MAGEA2B*, *MAGEA2*, and *MAGEA6*) on the X chromosome, which only had H3K4me3 peaks in HCT 116 cells (Supplementary Figure S12). As a reference, the intensities of the H3K4me3 peaks associated with *CETN2* and *NSDHL* were similar in all samples. The Long Intergenic Non-Protein Coding RNA 1812 (*LINC01812*) gene had a broad H3K4me3 domain in RKO cells but not in the other samples (Supplementary Figure S13). The Myeloma Overexpressed (*MYEOV*) gene was identified as having a broad H3K4me3 domain in all colorectal cancer cells but not in normal colon samples (Supplementary Figure S14). Taking a broader chromosome view shows that the two-pore segment channel 2 (*TPCN2*) gene had an H3K4me3 peak in the normal colon and colorectal cancer cell lines, whereas an H3K4me3 peak for the *MYEOV* gene was only observed in the colorectal cancer cell lines (Supplementary Figures S14 and S15). Collectively, our data provide a list of unique genes that could be used for designing new therapies for specifically targeting colorectal cancer cells.

## 3. Discussion

The H3K4me3-modified nucleosomes play multiple roles in the regulation of transcription. We capitalized on this feature of H3K4me3 to identify differentially marked genes in normal colon cells versus different types of colorectal cancer to identify genes essential to the biology of normal colon cells and those critical to specific colorectal cancer cells. Our high-resolution ChIP-sequencing data showed that most H3K4me3-marked genes were similar in normal colon cells and colorectal cancer cell lines. However, our analytical pipeline successfully identified genes with H3K4me3 peaks in normal, but not colorectal cancer cells. However, this analytical pipeline was less productive in identifying H3K4me3-marked genes in colorectal cancer cells. Although we are uncertain why this pipeline failed, we believe the MACS3 peak caller in Partek Flow was an issue. Nevertheless, the results suggest that multiple genes expressed in normal colon epithelial cells are silenced in colorectal cancer cells by well-documented processes such as promoter DNA methylation and repressive histone PTMs such as H3K27me3.

The unique genes marked with H3K4me3 in normal colon cells, but not colorectal cancer cells, were involved in cell adhesion and nervous system development. Of the 35 genes involved in the biological process of “homophilic cell adhesion via plasma membrane adhesion molecules”, 29 genes were confirmed to have an H3K4me3 peak in normal colon cells and not in colorectal cancer cells. Of the 56 genes involved in the biological process “cell adhesion”, 43 were confirmed to have an H3K4me3 peak only in the normal colon cells. Most colon epithelial genes involved in cell adhesion have narrow H3K4me3 peaks. The expression of these genes contributes to the differentiation of normal colon cells, which is inhibited in colorectal cancer cells [15]. Furthermore, we found that several genes coding for junctional adhesion molecules (JAM) (*JAM2* and *JAM3*) have H3K4me3 peaks in normal colon cells but not in colorectal cancer cells. One strategy for treating colorectal cancer patients is to apply epigenetic drugs, such as histone deacetylase inhibitors and protein arginine methyltransferase inhibitors (MS023), to induce the differentiation of colorectal cancer cells, referred to as differentiation therapy [15,16].

An unexpected result was that several genes with an H3K4me3 peak in normal colon cells but not in colorectal cancer cells were involved in the biological process “nervous system development”. Among the 47 genes involved in nervous cell development, 35, including *GRAF1*, BMP/retinoic acid-inducible neural-specific 2 (*BRINP2*), *NTF3*, endothelin receptor type B (*EDNRB*), erb-b2 receptor tyrosine kinase 4 (*ERBB4*), and protein kinase D1 (*PRKD1*), were confirmed to have H3K4me3 peaks in the sigmoid colon and CCD 841 CoN, but not in colorectal cancer cells. Most of these genes have narrow H3K4me3 domains. A few genes with broad H3K4me3 domains have essential roles in the colon. The endothelin receptor type B (*EDNRB*) gene, a potential tumor suppressor gene, is vital for developing the enteric nervous system in the colon and is often silenced in colorectal cancer [17,18]. The glial cell-derived neurotrophic factor (*GDNF*) gene develops in the enteric nervous system and plays a role in the intestinal epithelial barrier and wound healing [19,20].

*FOXF1* adjacent non-coding developmental regulatory RNA (*FENDRR*) is a tumor suppressor gene with a broad H3K4me3 domain found only in colon epithelial cells. FENDRR encodes a long non-coding RNA (lncRNA) that plays an essential role in the development of the gastrointestinal system [21]. The *FENDRR* lncRNA is associated with chromatin-modifying complexes, such as the repressive PRC2 complex, and may have a role in regulating the transcription of target genes [21,22]. FENDRR is often silenced in colorectal cancer [21,22].

The *ELFN1-AS1* lncRNA gene is among the genes with a broad H3K4me3 domain in colorectal cancer cells but not in normal colon cells. The *ELFN1-AS1* lncRNA interacts with repressive PRC2 and DNMT3A and targets the Meis Homeobox 1 (*MEIS1*) promoter region in colorectal cancer cells [23]. MEIS1, a potential tumor suppressor gene involved in differentiation, has a broad H3K4me3 domain in normal colon cells but not in colorectal cancer cells. The *MEIS1* gene has a narrow H3K4me3 peak in HT-29 colorectal cancer cells, whereas the gene in HCT116 and RKO cells was not associated with an H3K4me3 peak, suggesting that silencing of the *MEIS1* promoter by the *ELFN1-AS1* lncRNA is more acute in HCT116 and RKO cells.

ChIP-Seq and single-molecule imaging methods have been developed to detect plasma cell-free nucleosomes with histone PTMs such as H3K4me3 to detect cancer cell types (e.g., colorectal cancer cells) [24,25,26]. The identification of colorectal cancer cell-specific genes with the broad H3K4me3 domain will aid in the development of cell-free DNA assays for diagnosis of colorectal cancer using blood samples [25,26].

## 4. Materials and Methods

### 4.1. Cell Culture

The colorectal cancer cells (HCT 116, HT-29, and RKO) were cultured under the conditions recommended by ATCC (Manassas, VA, USA). HCT 116 cells (CCL-247) and HT-29 cells (HTB-38) were cultured in McCoy’s 5a medium supplemented with 10% FBS. RKO (CRL-2577) and CCD 841 CoN (CRL-1790) cells were cultured in Eagle’s Minimum Essential Medium supplemented with 10% FBS. The characteristics of the cell lines are listed in Supplementary Table S1.

### 4.2. Histone Isolation and Immunoblot Analysis

Histones were extracted (0.4 N H_2_SO_4_) from frozen cell pellets (colonic epithelial CCD 841 CoN and colorectal cancer cell lines) as described previously [27]. Histones were electrophoretically resolved using 15% SDS-PAGE and transferred to nitrocellulose membranes. The membranes were stained with Ponceau S for two min, followed by destaining with TBST (2 mM Tris Base, 15 mM NaCl, 0.1% Tween-20, pH 7.6). The membranes were immunochemically stained with an anti-H3K4me3 antibody (Abcam, Cambridge, MA, USA), ab8580, lot# GR273043-1).

### 4.3. Chromatin Immunoprecipitation (ChIP) Assays and Sequencing

The cells were crosslinked with formaldehyde as described previously [28]. Crosslinked chromatin was digested with micrococcal nuclease to the extent that 70% of the chromatin was cleaved into mononucleosomes (Supplementary Figure S2). Before performing the ChIP assay, the specificity of the anti-H3K4me3 antibody (Millipore Sigma (Oakville, ON, Canada) 17-614 Lot# 2871690) was evaluated. Immunodot blot analysis demonstrated that the antibody detected only H3 peptides with K4me3 (Supplementary Figure S3). The antibody efficiently immunoprecipitated crosslinked mononucleosomes with H3K4me3 (Supplementary Figure S4). ChIP assays with the anti-H3K4me3 antibody and a control rabbit IgG were performed to produce input and ChIP fractions from the four cell lines to determine the enrichment of H3K4me3 mononucleosomes associated with the *MCL1* exon 1, exon 2, exon 3, and intron 1. Twelve A_260_ of each cell lysate were used for immunoprecipitation, and A_260_ was used as the input. The primer sequences are shown in Supplementary Table S1 of [28]. Supplementary Figure S5 shows an example of enrichment obtained in the ChIP assay using the anti-H3K4me3 antibody.

ChIP libraries were prepared using a NEBNext Ultra II DNA Library Prep Kit (Illumina, San Diego, CA, USA). The libraries were sequenced in Genome Quebec (Montréal, QC, Canada) using the Illumina HiSeq 4000 PE100 System at 25 million reads.

### 4.4. Partek Flow Analyses

BAM files from Genome Quebec were aligned with the human assembly GRCh37/hg19. The Partek flow pipeline included the following steps: (1) convert alignments to unaligned reads, (2) filter out contaminants (rDNA and mtrDNA), (3) pre-align QA/QC (Supplementary Table S2), (4) align reads to human assembly GRCh38/hg38 using Bowtie 2, (5) peak call using MACS3 (default or broad setting), and annotate peaks using Ensembl Transcripts release 91 (TSS region upstream and downstream limits set to 1000 bp). The tracks were produced using the Partek Chromosome View.

### 4.5. Data Collection

FASTQ files for the human normal colon were processed using the Partek Flow pipeline. The samples were *Homo sapiens* sigmoid colon tissue from a male child (3 years) (GEO: GSM956024 and GSM1227072), and *Homo sapiens* transverse colon tissue from a female adult (53 years) (GEO: GSE101371).

## 5. Conclusions

In summary, mapping H3K4me3 narrow and broad configurations identified genes with differential functions in normal colon and colorectal cancer cells. Our results will contribute to developing plasma cell-free DNA assays for screening colorectal cancer. We believe that an improvement in identifying differential histone PTM peaks will be an important tool for identifying genes with different functions in normal and diseased cells.

## Figures and Tables

**Table 1:** GO terms (biological processes) for genes with H3K4me3 peaks in CCD 841 CoN and sigmoid colon and not in colorectal cancer cells.

Biological Process (Ensembl)	Benjamini Score
Homophilic cell adhesion via plasma membrane adhesion molecules	5.6 × 10^−11^
Cell adhesion	5.5 × 10^−9^
Nervous system development	5.3 × 10^−8^
Axon guidance	3.9 × 10^−6^
Potassium ion transmembrane transport	1.7 × 10^−4^

## Data Availability

The sequencing data are available from GEO under accession number GSE286296.

## Supplementary Materials

The following supporting information can be downloaded at: https://www.mdpi.com/article/10.3390/ijms26062546/s1.
