# Supplementary material for: Differential H3K4me3 Domains in Normal and Colorectal Cancer Cells Reveal Novel Epigenetic Targets
Source: Int J Mol Sci. 2025 Mar 12;26(6):2546. doi: 10.3390/ijms26062546 (PMC11942224; doi:10.3390/ijms26062546)
Supplement: Supplementary file 1 [file ijms-26-02546-s001.zip › ijms-3499151-supplementary.pdf]

Figure S1

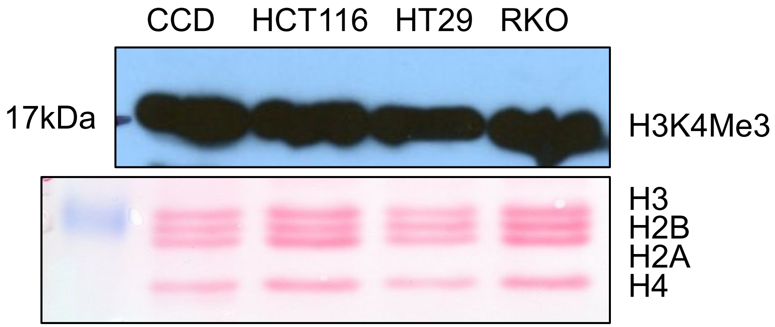

Figure S1. Immunoblot analysis of H3K4me3 levels in normal colon epithelial and different colorectal cancer cell lines. Total histones were acid extracted from nuclei isolated from frozen cell pellets. The histones were resolved by SDS PAGE and transferred to nitrocellulose. The blot was stained with Ponceau S (lower panel) and immunochemically stained with an H3K4me3 antibody (ab8580; lot# GR273043-1) and ECL (upper panel).

Figure S2

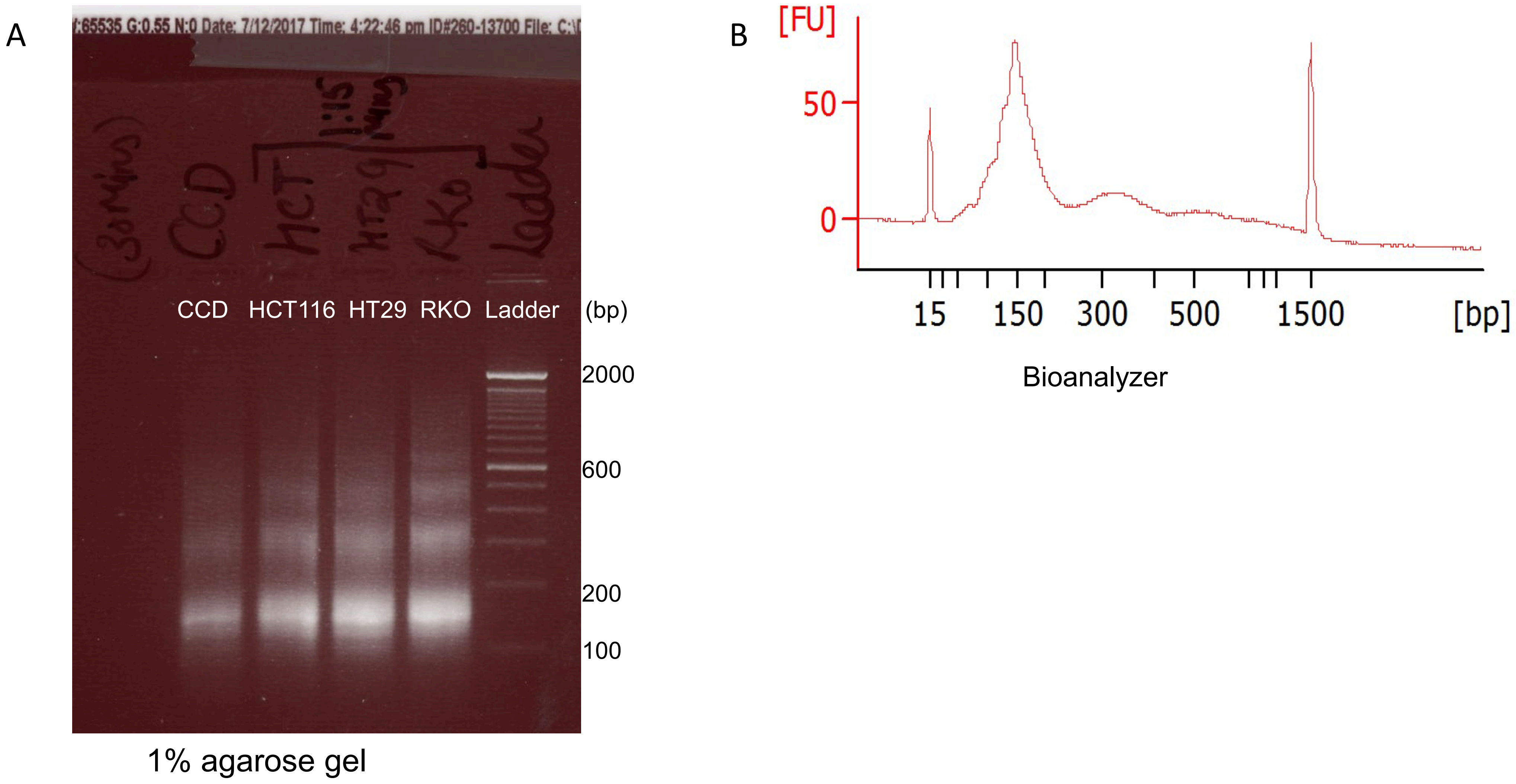

Figure S2. Micrococcal digestion and quantification of mononucleosomes in normal colon epithelial and different colorectal cancer cell lines. Nuclei from the cell lines was digested to yield 70% mononucleosomes. Panel A shows the sizes of the DNA fragments after micrococcal nuclease digestion. Panel B shows a typical DNA size distribution after micrococcal digestion.

Figure S3

Dot blot using Milipore H3K4me3 antibody

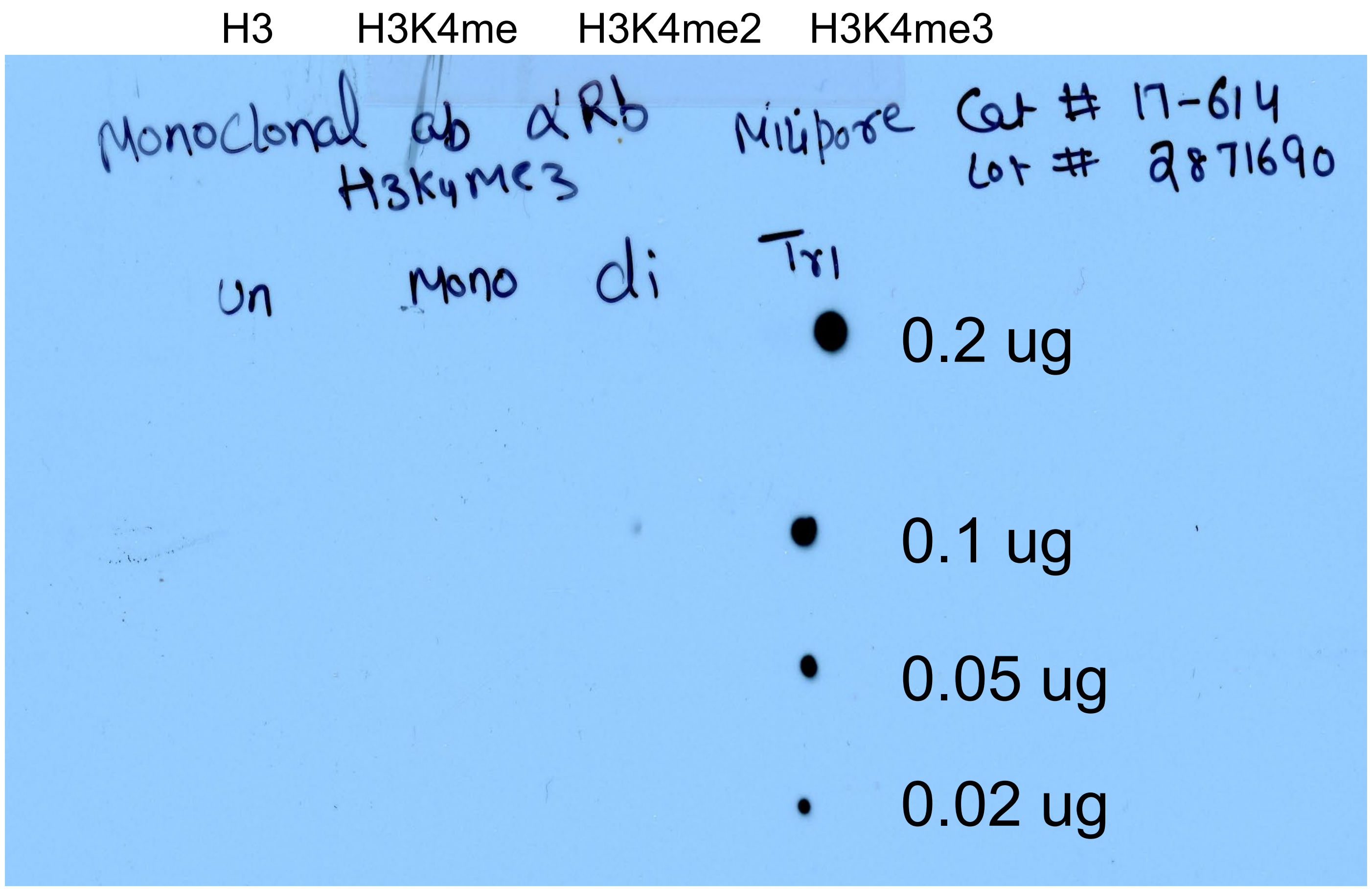

Primary ab 1:2000 dilution
Secondary ab 1:5000 dilution

Figure S3. Dot blot assay for confirming specificity of H3K4me3 antibody. The anti-H3K4me3 antibody (Millipore Sigma 17-614 Lot# 2871690) recognition of H3 peptides that were unmodified or had K4me1, K3me4, and K4me3 were tested by dot immunoblots. The amount of peptide added to the blot is indicated.

Figure S4

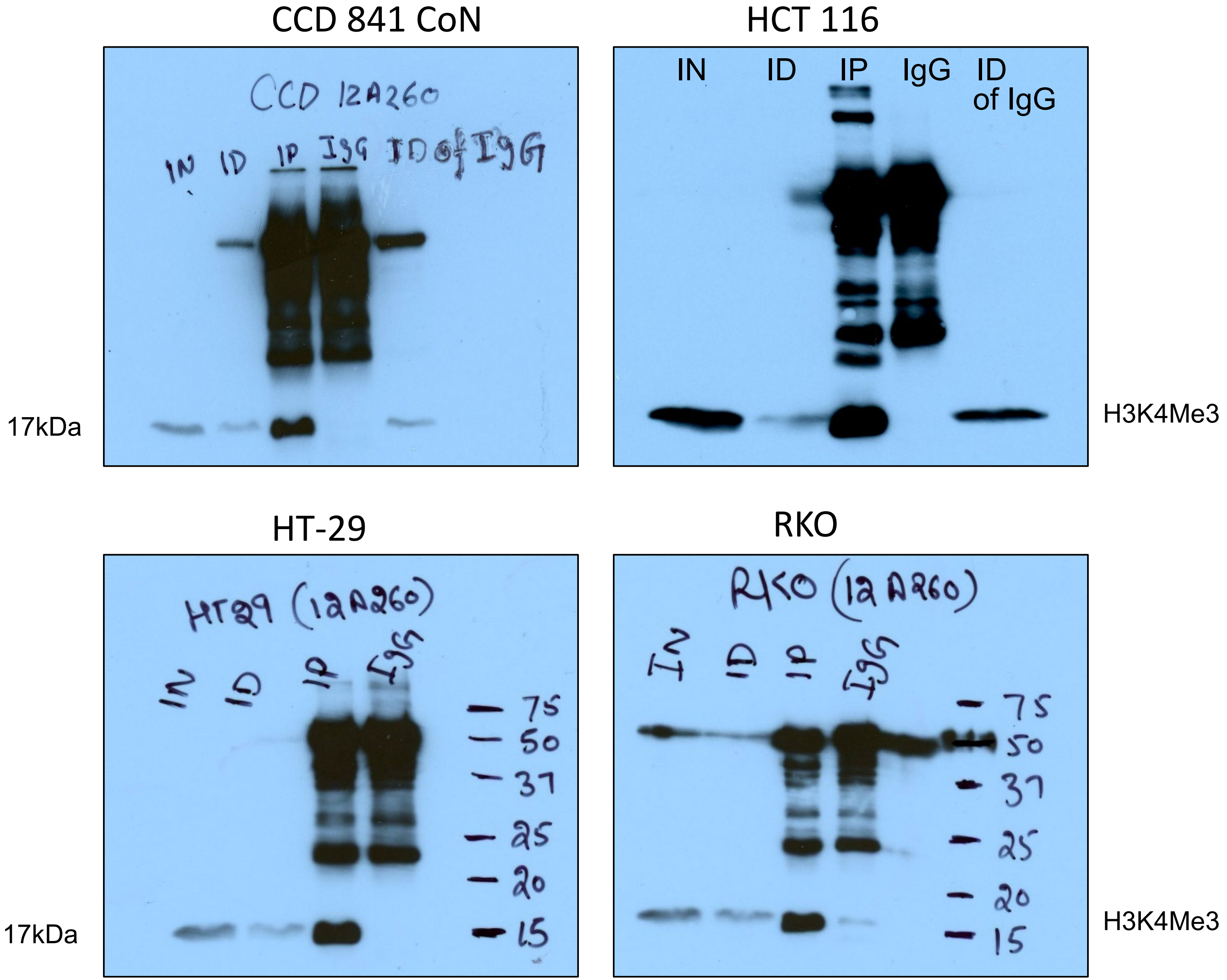

Figure S4. H3K4me3 chromatin immunoprecipitation efficiency assessed in samples from normal colon epithelial and different colorectal cancer cell lines. Immunoprecipitation efficiency test with anti-H3K4me3 antibody and cell lysates. The cell line is indicated at the top of each panel. IN, input; ID, immunodepleted; IP, immunoprecipitated.

Figure S5

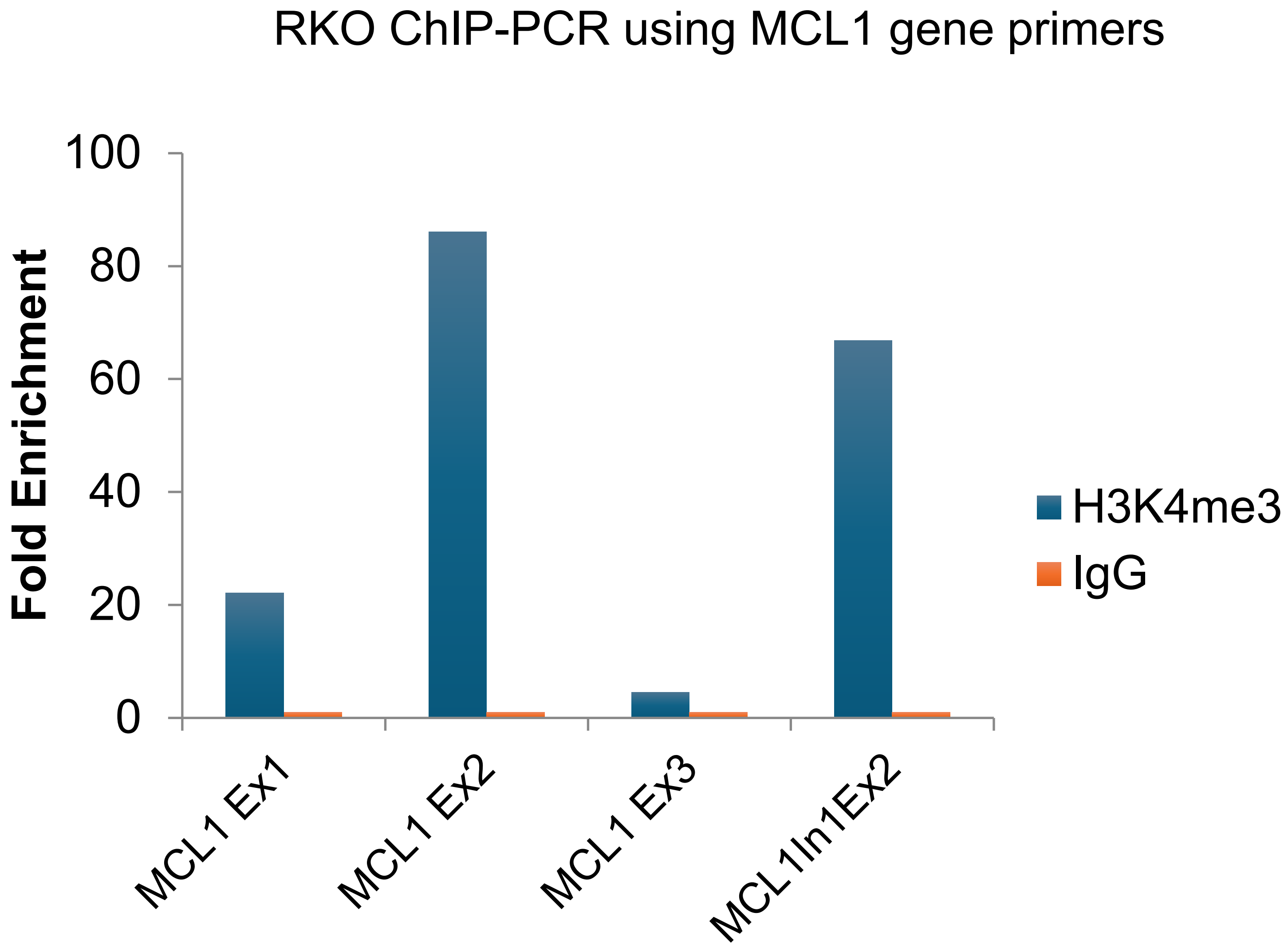

Figure S5. ChIP-PCR showing the fold enrichment of H3K4me3 at the mononucleosomes associated with the *MCL1* exon 1, exon 2, exon 3, and intron 1. Enrichment of *MCL1* gene body sections H3K4me3-ChIP DNA relative to input DNA from RKO cells was determined by PCR using primers to exon 1 (MCL1 Ex1), exon 2 (MCL1 Ex2), exon 3 (MCL1 Ex3), and intron 1-exon 2 (MCL1IN1Ex2). A control ChIP was done with rabbit IgG.

Figure S6

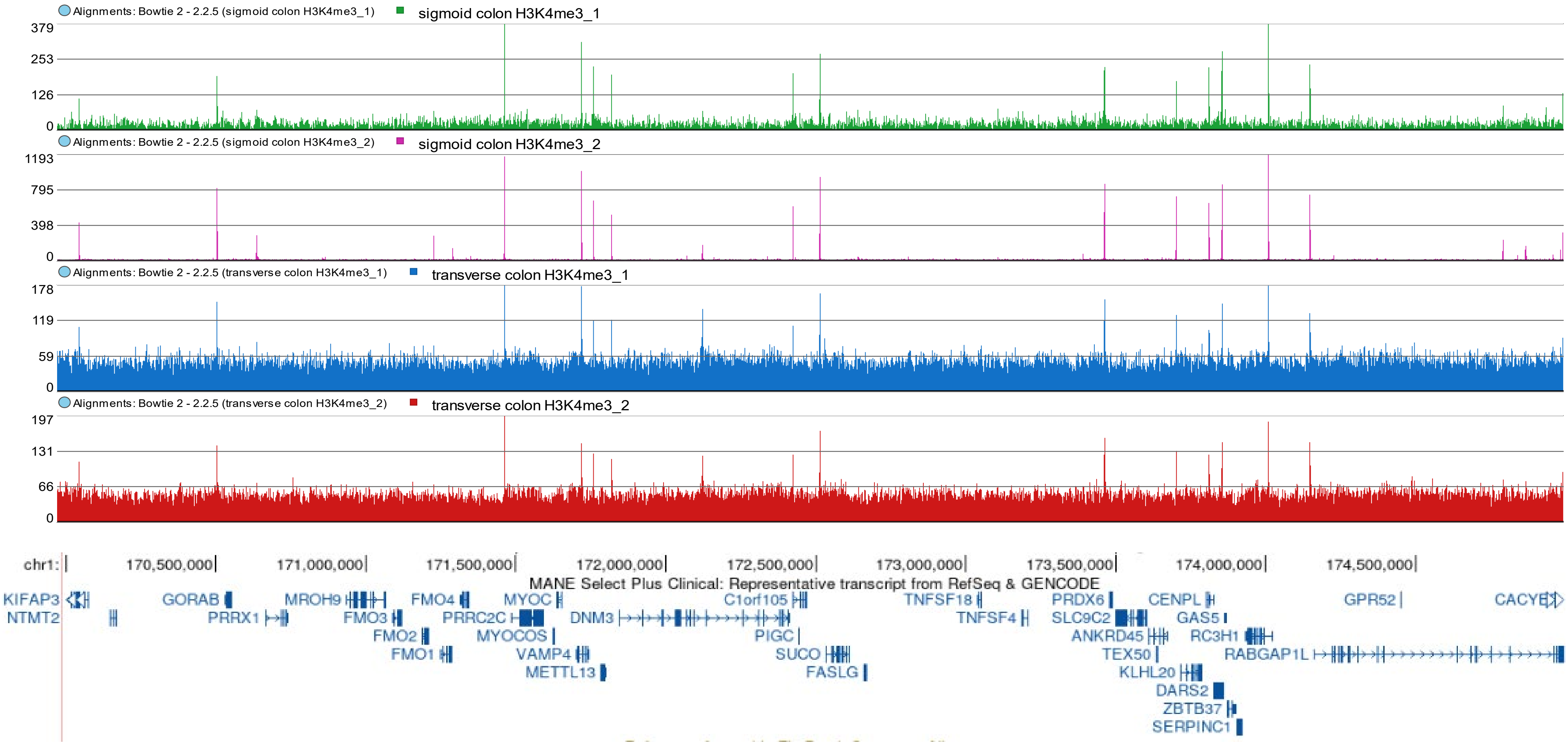

Figure S6. H3K4me3 chromatin immunoprecipitation sequencing tracks for chromosome 1:170,500,000 to 174,500,000 in sigmoid colon (sigmoid colon H3K4me3_1, SRR518490; sigmoid colon H3K4me3_2, SRR1045811) and transverse colon (transverse colon H3K4me3_1, SRR5833166; transverse colon H3K4me3_2 , SRR5833167).

Figure S7

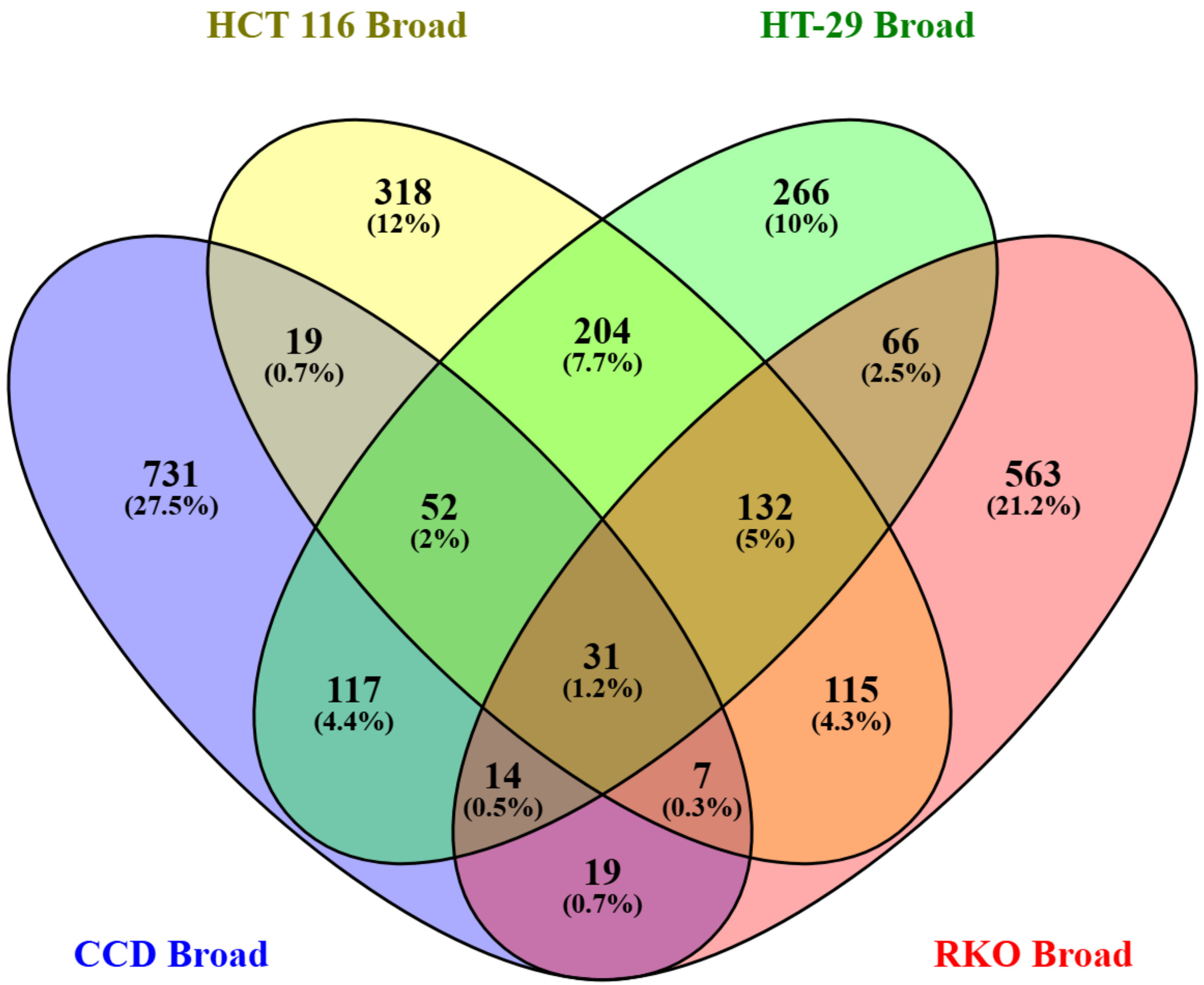

Figure S7. Venn diagram showing the H3K4me3 overlapping and unique broad domains in CCD 841 CoN, HCT 116, HT-29 and RKO cell lines. The distribution of genes with an H3K4me3 peak using the MACS3 broad setting in CCD 841 CoN (normal colon) and colorectal cancer cells (HCT116, HT-29, RKO).

Figure S8

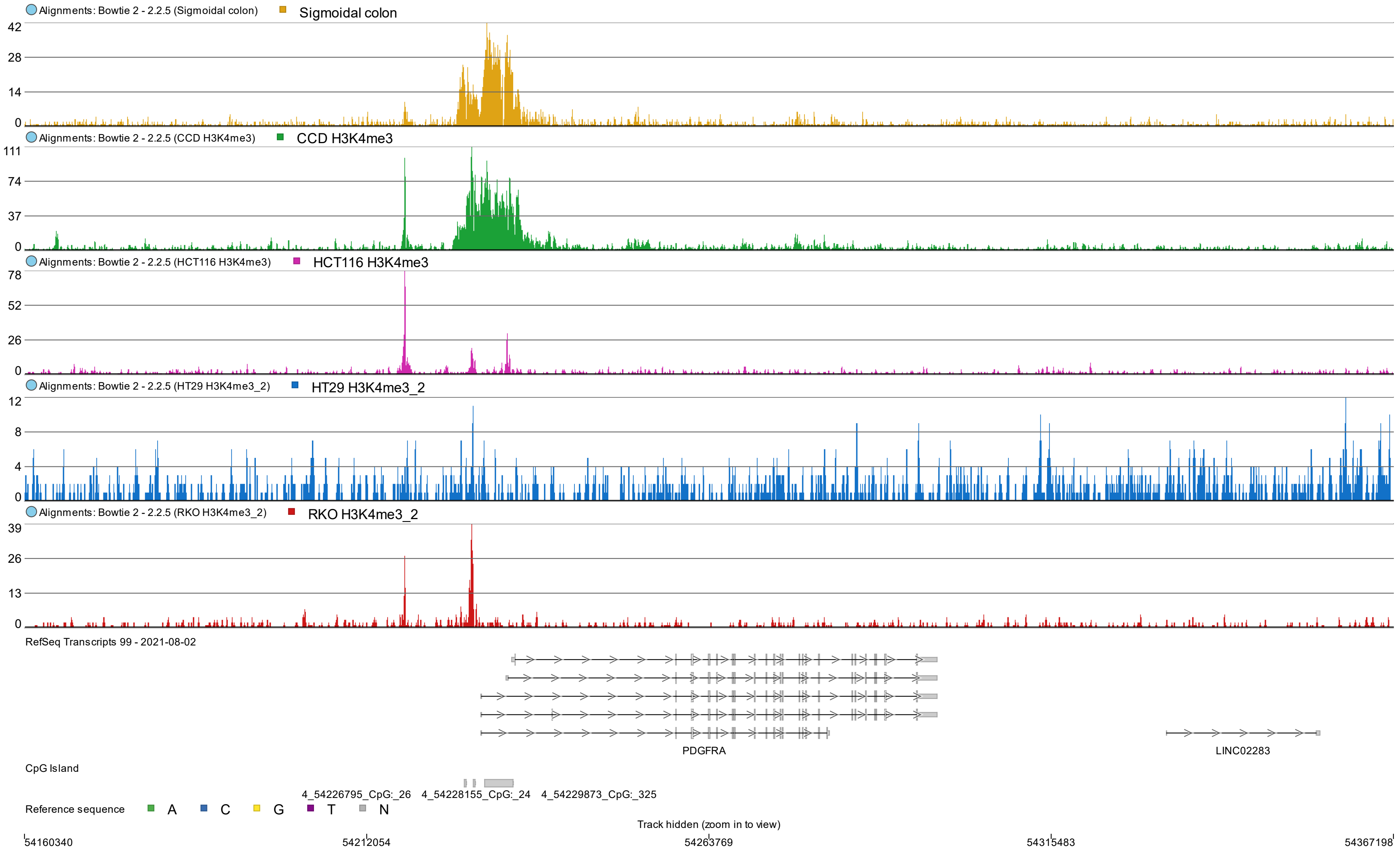

Figure S8. Gene track of platelet-derived growth factor receptor alpha (*PDGFRA*) showing H3K4me3 broad domain in CCD 841 CoN relative to HCT 116, HT-29 and RKO cell lines. H3K4me3 ChIP-Seq track for the *PDGFRA* gene in sigmoid colon (SRR1045811), CCD 841 CoN (normal colon), and colorectal cancer cells (HCT 116, HT-29, RKO). Positions of CpG islands are indicated.

Figure S9

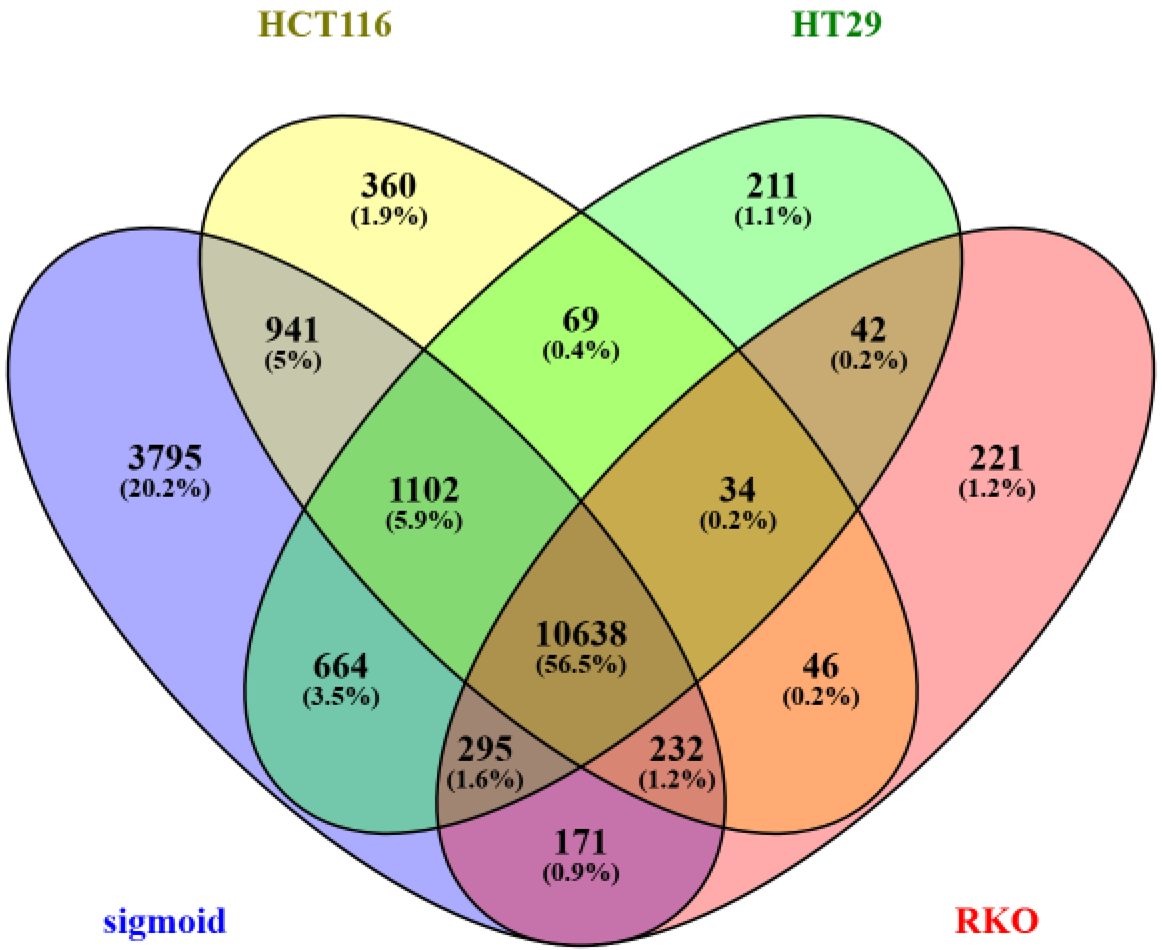

Figure S9. Venn diagram showing the H3K4me3 peaks in the Sigmoid colon tissue and colon cancer cell lines, HCT 116, HT-29 and RKO. The distribution of genes with an H3K4me3 peak using the MACS3 broad setting (no input) in sigmoid colon (SRR1045811) and colorectal cancer cells (HCT 116, HT-29, RKO).

Figure S10

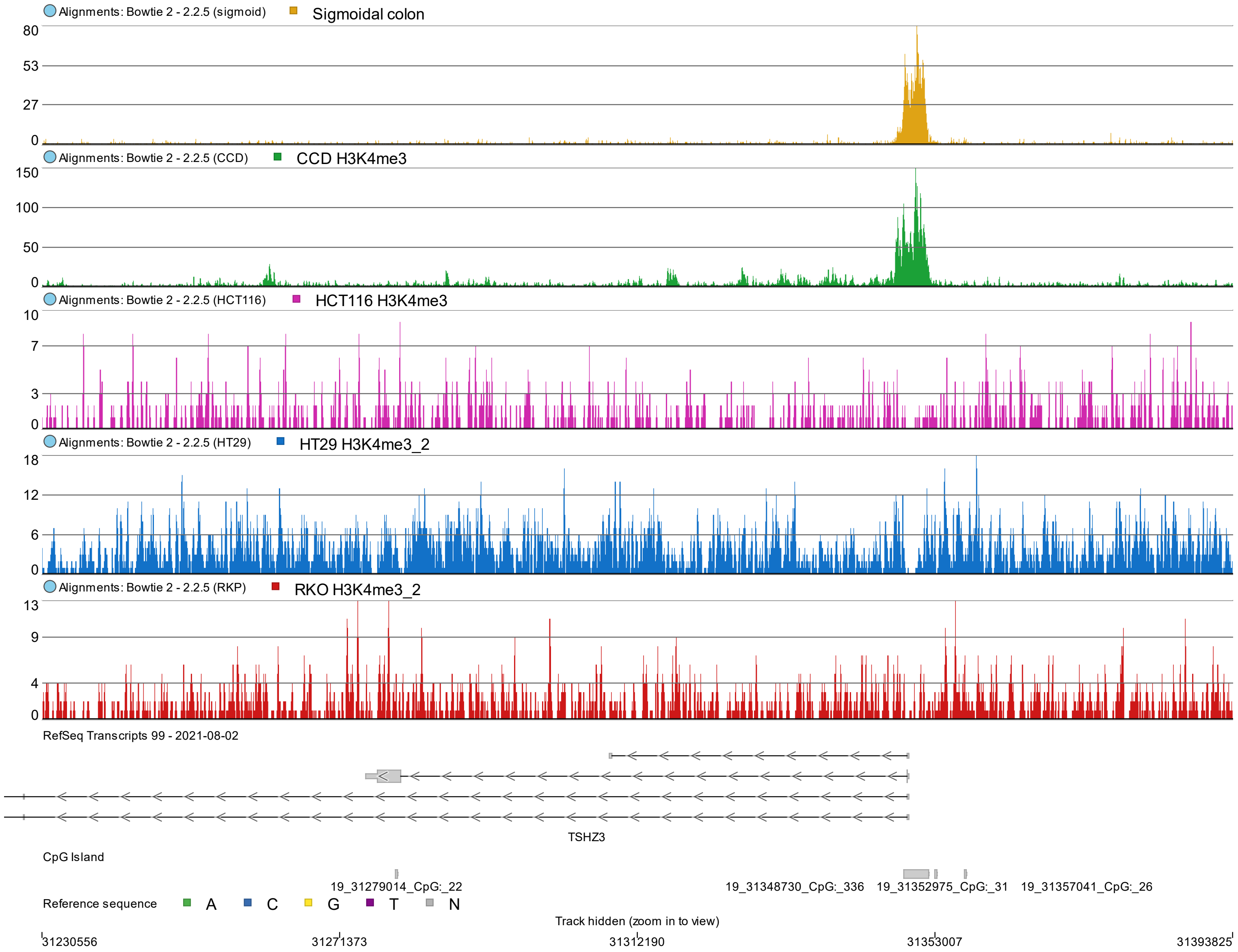

Figure S10. Teashirt Zinc Finger Homeobox 3 (*TSHZ3*) gene track showing H3K4me3 peak in the Sigmoid colon tissue and CCD 841 CoN cells relative to HCT 116, HT-29 and RKO cell lines. H3K4me3 ChIP-Seq track for the *TSHZ23* gene in sigmoid colon (SRR1045811), CCD 841 CoN (normal colon), and colorectal cancer cells (HCT 116, HT-29, RKO). Positions of CpG islands are indicated.

Figure S11

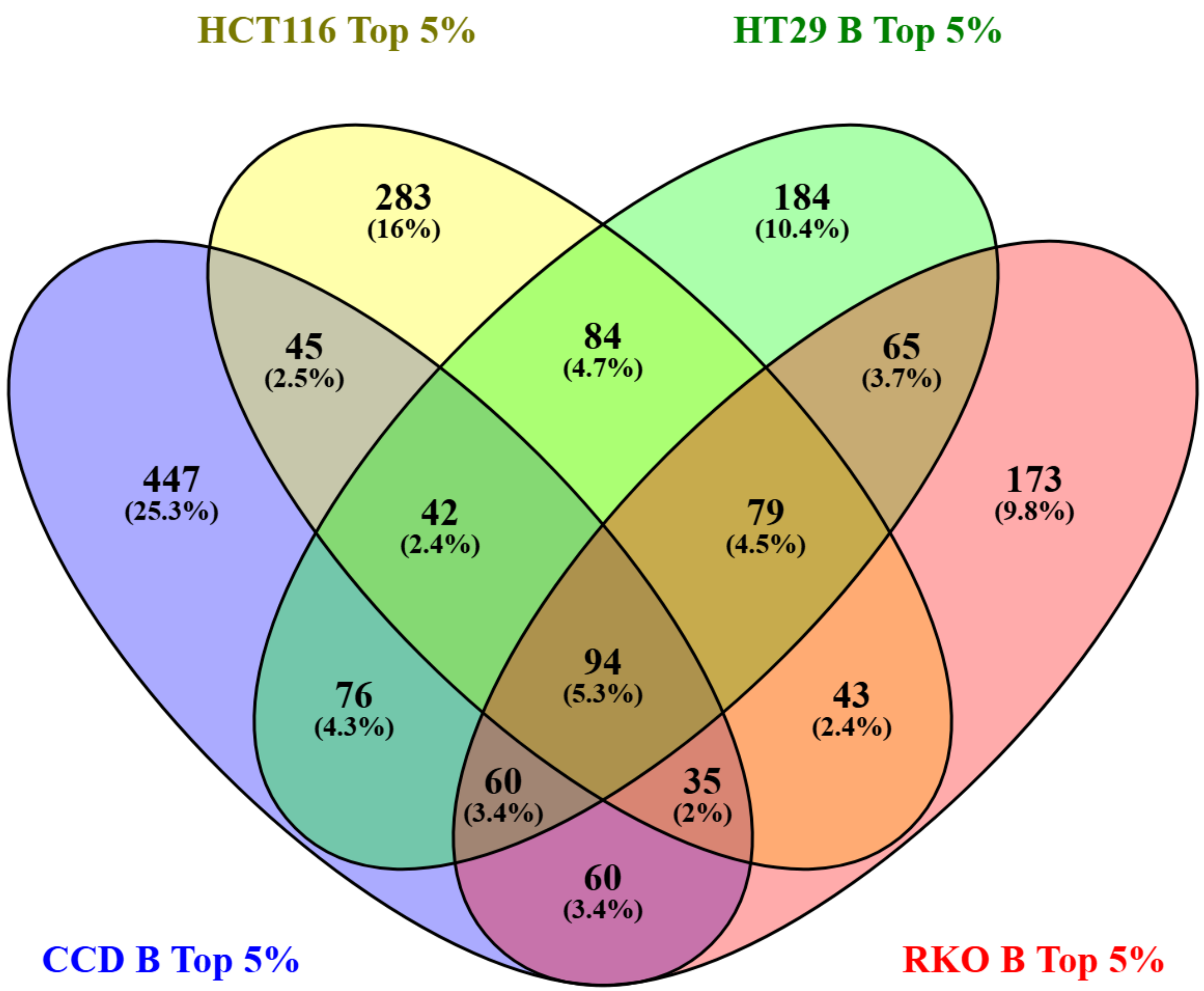

Figure S11. Top 5% genes with broad H3K4me3 domains with a TSS in the sigmoid colon tissue and CCD 841 CoN, HCT 116, HT-29, RKO cell lines. The distribution of genes with the top 5% broadest H3K4me3 domains identified by using the MACS3 broad setting (no input) in CCD 841 CoN (normal colon) and colorectal cancer cells (HCT 116, HT-29, RKO).

Figure S12

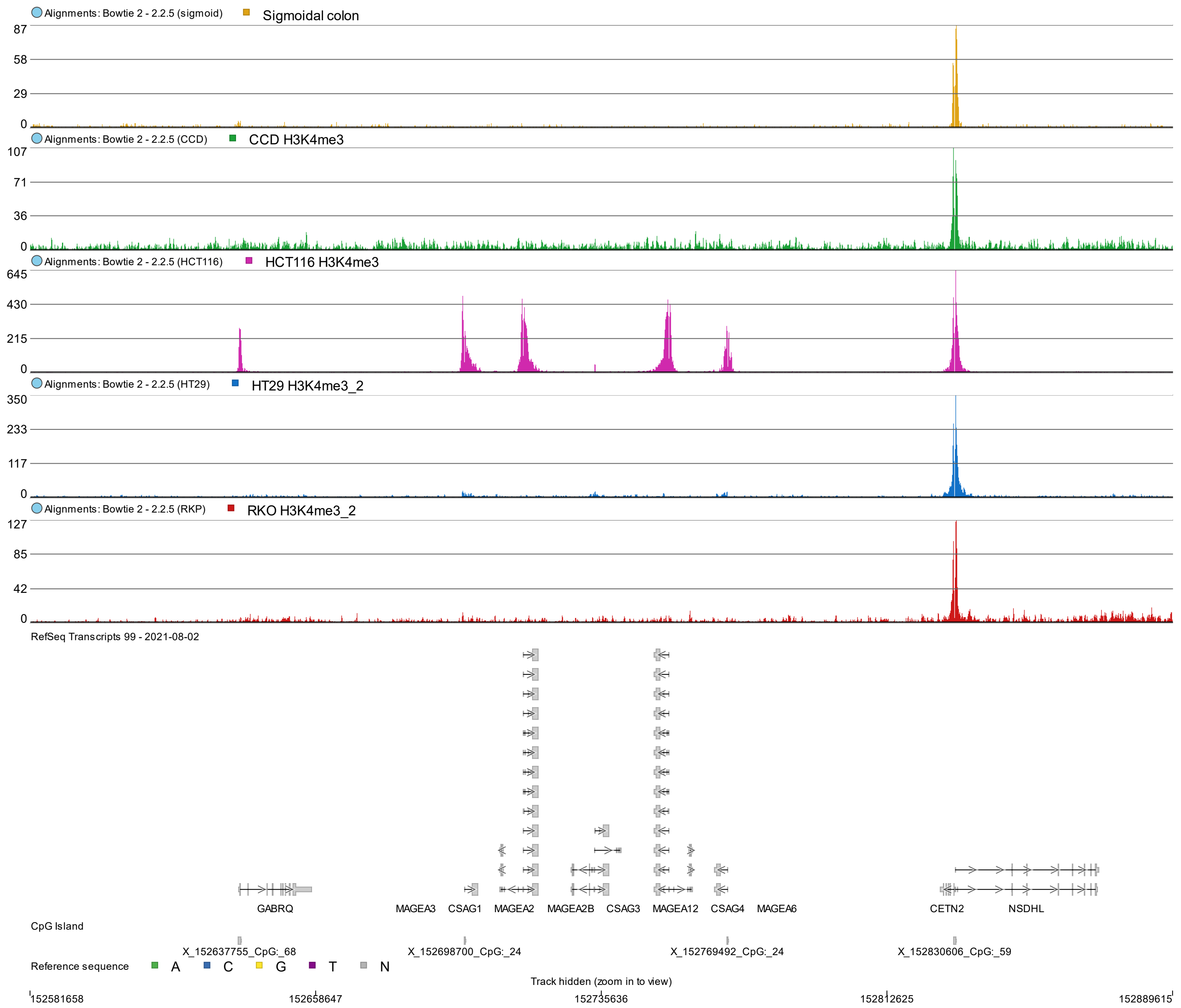

Figure S12. MAGE Family Member A2 (MAGEA2) gene track showing the H3K4me3 peaks only in HCT116 cells. H3K4me3 ChIP-Seq track for the cluster of *MAGEA* genes in sigmoid colon (SRR1045811), CCD 841 CoN (normal colon), and colorectal cancer cells (HCT 116, HT-29, RKO). Positions of CpG islands are indicated.

Figure S13

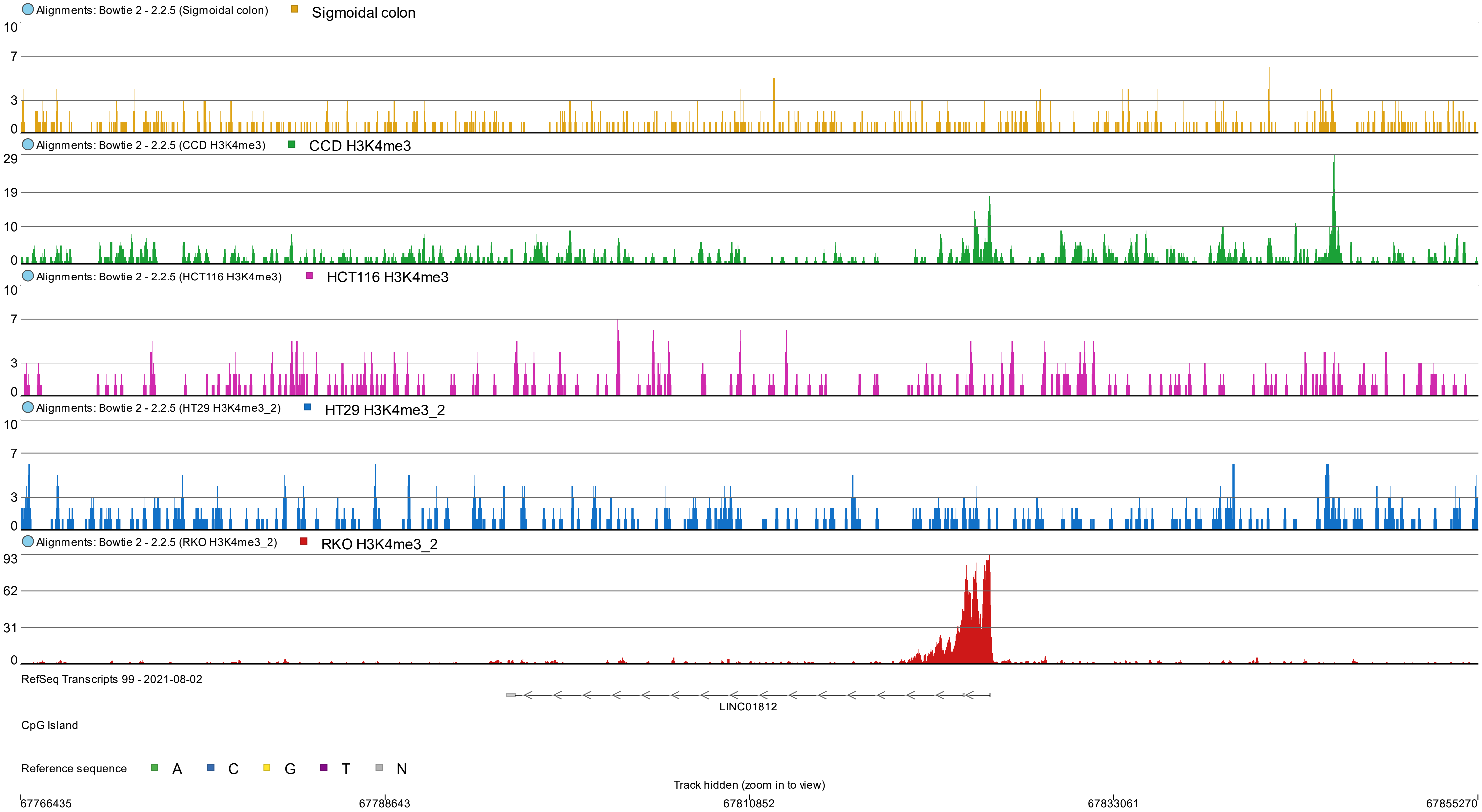

Figure S13. The Long Intergenic Non-Protein Coding RNA 1812 (*LINC01812*) gene track showing a broad H3K4me3 domain in RKO cells. H3K4me3 ChIP-Seq track for the *MLINC01812* gene in sigmoid colon (SRR1045811), CCD 841 CoN (normal colon), and colorectal cancer cells (HCT 116, HT-29, RKO). Positions of CpG islands are indicated.

Figure S14

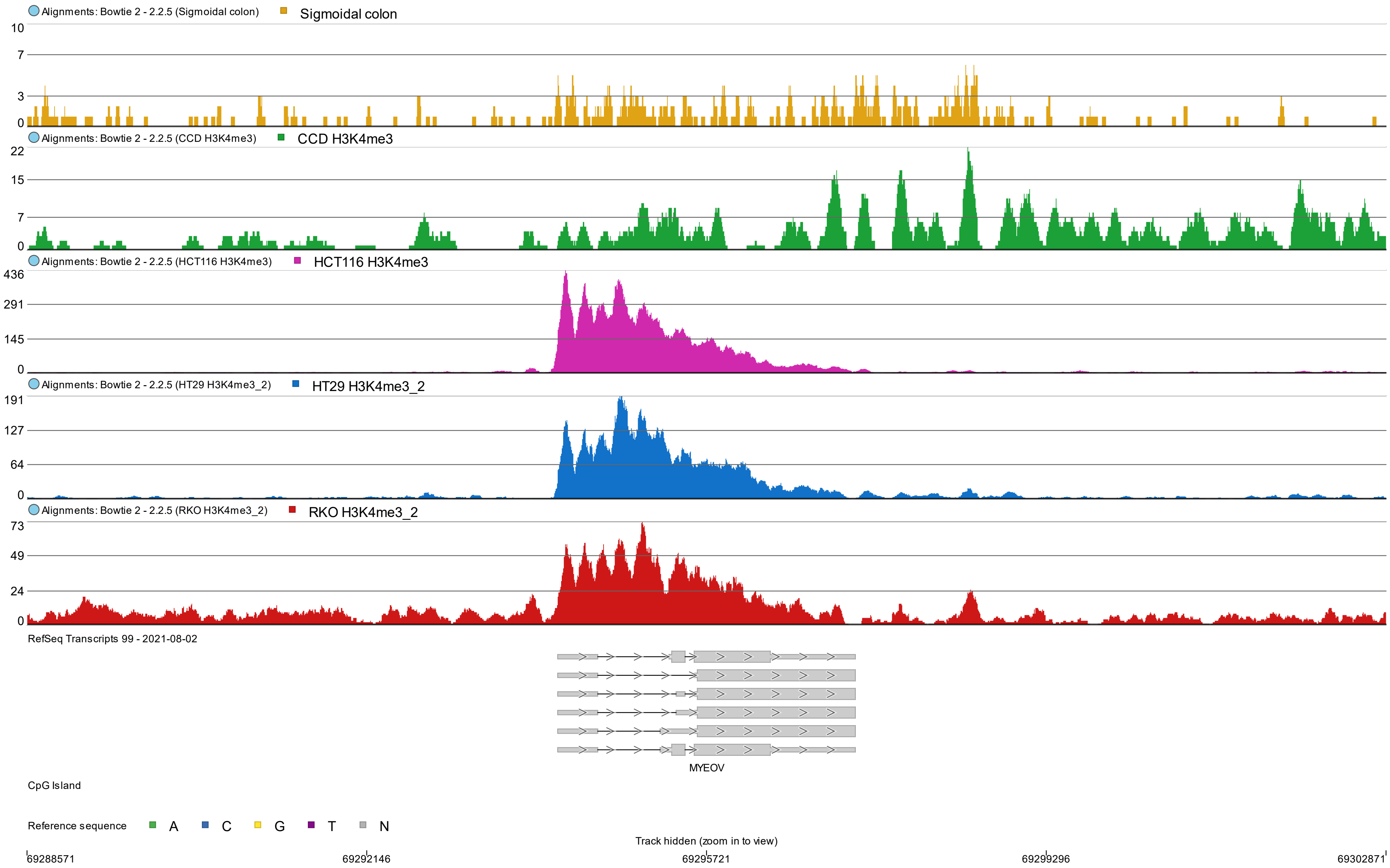

Figure S14. H3K4me3 ChIP-Seq track for the *MYEOV* gene in CCD 841 CoN (normal colon) and colorectal cancer cells (HCT116, HT-29, RKO).

Figure S15

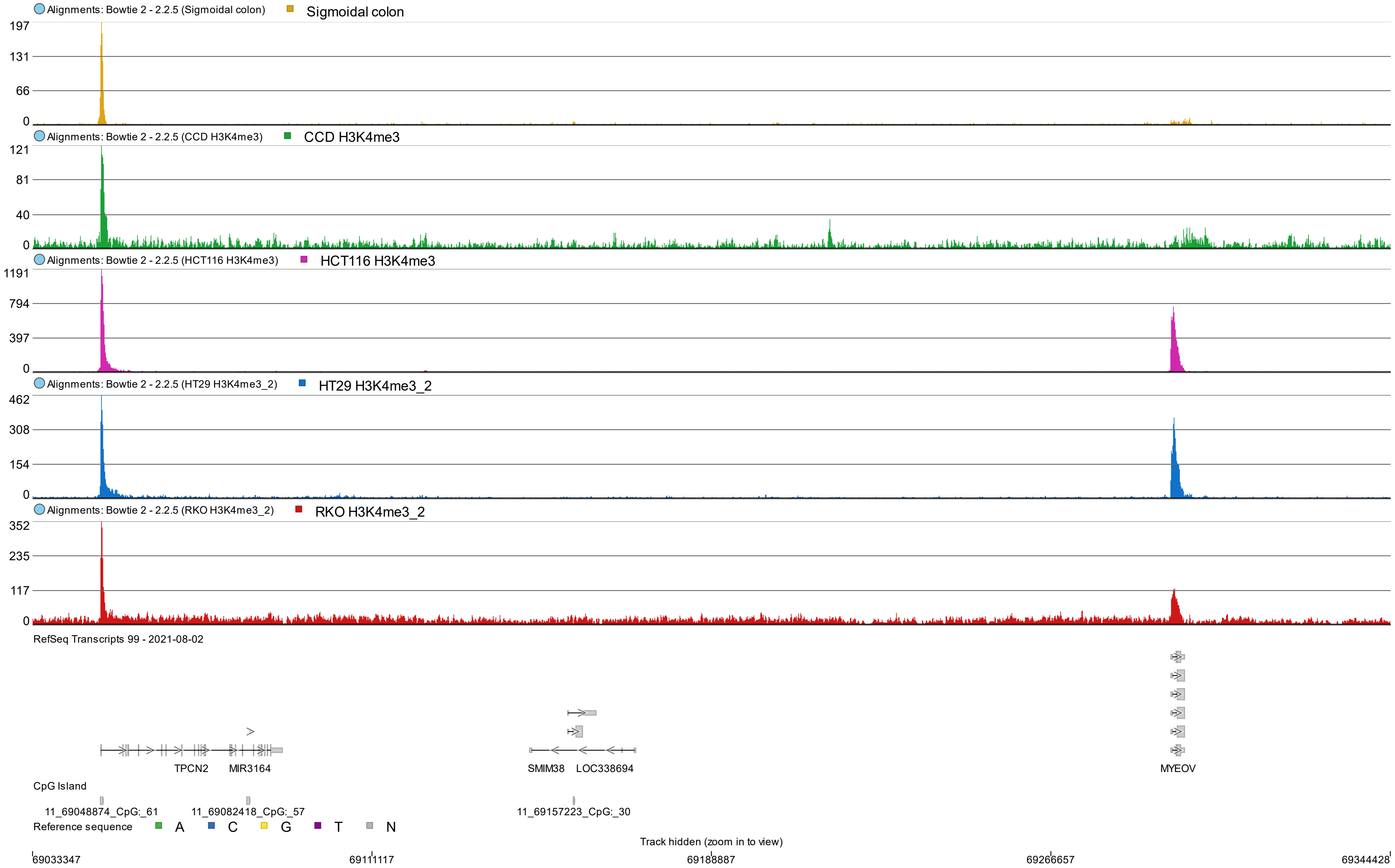

Figure S15. Gene tracks for Two Pore Segment Channel 2 (*TPCN2*) and Myeloma Overexpressed (*MYEOV*) genes showing H3K4me3 peaks in the sigmoid colon tissue, CCD 841 CoN, HCT 116, HT-29, RKO cell lines. H3K4me3 ChIP-Seq track for the chromosome region containing the *TPCN2* and *MYEOV* genes in sigmoid colon (SRR1045811), CCD 841 CoN (normal colon), and colorectal cancer cells (HCT 116, HT-29, RKO). Positions of CpG islands are indicated.

Table S1. Characteristics of cell lines

Name	Morph.	Tissue	Disease	Age	Sex	BRAF	KRAS	TP53	CIN	MSI
CCD 841 CoN	epithelial	Large intestine Colon	normal	21 weeks gestation	Female	WT	WT	WT	No	No
HCT 116	epithelial	Large intestine Colon	colorectal carcinoma	48 years old	Male	WT	G13D	WT	No	Yes
HT-29	epithelial	Colon	colorectal adeno carcinoma	44 years old	Female	V600E	WT	R273H	Yes	No
RKO	epithelial	Large intestine Colon	colorectal carcinoma	Not known	Female	V600E	WT	WT	No	Yes

Table S2. Pre-alignment QA/QC

Sample name	Total reads	Avg. read length	Avg. read quality	% N	% GC
CCD H3K4me3	23,803,634	99.91	38.76	0.01%	48.47%
CCD input	30,419,580	99.76	38.67	0.02%	43.85%
HCT116 H3K4me3	32,942,982	99.96	37.81	0.01%	56.94%
HCT116 input	24,827,946	99.89	38.92	0.01%	43.30%
HT29 H3K4me3_1	25,020,654	99.90	38.80	0.01%	49.31%
HT29 H3K4me3_2	22,756,469	99.85	38.10	0.01%	50.93%
HT29 input	26,692,410	99.92	38.81	0.01%	43.13%
RKO H3K4me3_1	27,789,360	99.90	38.68	0.01%	50.01%
RKO H3K4me3_2	25,416,734	99.92	38.17	0.01%	54.15%
RKO input	26,812,033	99.94	38.22	0.01%	43.22%

Table S3. GO terms (nervous system development, see Table 1)) are shown for genes with H3K4me3 peaks in the normal colon epithelial cells absent in the colon cancer cell lines.

ID	Gene Name	Track check
BRINP2	BMP/retinoic acid inducible neural specific 2(BRINP2)	yes
GFRA1	GDNF family receptor alpha 1(GFRA1)	yes
GFRA2	GDNF family receptor alpha 2(GFRA2)	yes
GFRA3	GDNF family receptor alpha 3(GFRA3)	yes
INSC	INSC spindle orientation adaptor protein(INSC)	yes
LDB2	LIM domain binding 2(LDB2)	yes
MDGA2	MAM domain containing glycosylphosphatidylinositol anchor 2(MDGA2)	yes
POU3F2	POU class 3 homeobox 2(POU3F2)	yes
RFNG	RFNG O-fucosylpeptide 3-beta-N-acetylglucosaminyltransferase(RFNG)	no
SIM1	SIM bHLH transcription factor 1(SIM1)	yes
SPOCK1	SPARC (osteonectin), cwcv and kazal like domains proteoglycan 1(SPOCK1)	yes
APBA2	amyloid beta precursor protein binding family A member 2(APBA2)	yes
CNTFR	ciliary neurotrophic factor receptor(CNTFR)	Yes ?
CRMP1	collapsin response mediator protein 1(CRMP1)	yes?
CNTN4	contactin 4(CNTN4)	yes
DLX6	distal-less homeobox 6(DLX6)	yes
DCLK1	doublecortin like kinase 1(DCLK1)	yes
EDNRB	endothelin receptor type B(EDNRB)	yes and broad
ERBB4	erb-b2 receptor tyrosine kinase 4(ERBB4)	yes
FGF13	fibroblast growth factor 13(FGF13)	yes
FGF5	fibroblast growth factor 5(FGF5)	yes
GDNF	glial cell derived neurotrophic factor(GDNF)	yes and broad
GLRB	glycine receptor beta(GLRB)	yes
ITGA8	integrin subunit alpha 8(ITGA8)	yes
JAG1	jagged canonical Notch ligand 1(JAG1)	no
MAB21L2	mab-21 like 2(MAB21L2)	yes and broad
NEXMIF	neurite extension and migration factor(NEXMIF)	yes
NEUROG1	neurogenin 1(NEUROG1)	no
NTF3	neurotrophin 3(NTF3)	yes
NDP	norrin cystine knot growth factor NDP(NDP)	yes
PTN	pleiotrophin(PTN)	yes
PCSK2	proprotein convertase subtilisin/kexin type 2(PCSK2)	yes
PRKD1	protein kinase D1(PRKD1)	yes intense
PCDH10	protocadherin 10(PCDH10)	yes
PCDHA4	protocadherin alpha 4(PCDHA4)	?
PCDHB1	protocadherin beta 1(PCDHB1)	yes
PCDHB15	protocadherin beta 15(PCDHB15)	no
PCDHB2	protocadherin beta 2(PCDHB2)	no
PCDHB5	protocadherin beta 5(PCDHB5)	no
PCDHB7	protocadherin beta 7(PCDHB7)	yes
PCDHGA11	protocadherin gamma subfamily A, 11(PCDHGA11)	yes
PCDHGA7	protocadherin gamma subfamily A, 7(PCDHGA7)	yes
PCDHGB4	protocadherin gamma subfamily B, 4(PCDHGB4)	yes
PCDHGB5	protocadherin gamma subfamily B, 5(PCDHGB5)	yes
PCDHGB7	protocadherin gamma subfamily B, 7(PCDHGB7)	yes
TCF4	transcription factor 4(TCF4)	yes
ZEB2	zinc finger E-box binding homeobox 2(ZEB2)	yes

Cell adesion 56 genes

ID	Gene Name	
ADAM12	ADAM metallopeptidase domain 12(ADAM12)	yes
ADAM23	ADAM metallopeptidase domain 23(ADAM23)	yes
CCL2	C-C motif chemokine ligand 2(CCL2)	no
CXCL12	C-X-C motif chemokine ligand 12(CXCL12)	yes and broad
EPHA3	EPH receptor A3(EPHA3)	yes
HCK	HCK proto-oncogene, Src family tyrosine kinase(HCK)	no
RIPOR2	RHO family interacting cell polarization regulator 2(RIPOR2)	yes
SPOCK1	SPARC (osteonectin), cwcv and kazal like domains proteoglycan 1(SPOCK1)	yes
ACTN2	actinin alpha 2(ACTN2)	yes but may have a different start site in the three CRC lines
ACKR3	atypical chemokine receptor 3(ACKR3)	yes
CDH11	cadherin 11(CDH11)	yes and intense
CDH2	cadherin 2(CDH2)	yes and intense
CDH6	cadherin 6(CDH6)	yes
CDHR1	cadherin related family member 1(CDHR1)	yes
CHST10	carbohydrate sulfotransferase 10(CHST10)	yes
CHL1	cell adhesion molecule L1 like(CHL1)	yes and intense
CLDN11	claudin 11(CLDN11)	yes
COL6A3	collagen type VI alpha 3 chain(COL6A3)	yes
DSG2	desmoglein 2(DSG2)	no
DDR2	discoidin domain receptor tyrosine kinase 2(DDR2)	yes
EFS	embryonal Fyn-associated substrate(EFS)	yes
IGFBP7	insulin like growth factor binding protein 7(IGFBP7)	yes
ITGA11	integrin subunit alpha 11(ITGA11)	yes
LAMA1	laminin subunit alpha 1(LAMA1)	yes
LAMA2	laminin subunit alpha 2(LAMA2)	yes
NELL2	neural EGFL like 2(NELL2)	yes

NCAM1	neural cell adhesion molecule 1(NCAM1)	yes
NCAM2	neural cell adhesion molecule 2(NCAM2)	yes
NTM	neurotrimin(NTM)	yes
NID2	nidogen 2(NID2)	yes
PDPN	podoplanin(PDPN)	yes
PSTPIP1	proline-serine-threonine phosphatase interacting protein 1(PSTPIP1)	no
PTPRD	protein tyrosine phosphatase receptor type D(PTPRD)	yes
PCDH10	protocadherin 10(PCDH10)	yes
PCDH17	protocadherin 17(PCDH17)	yes
PCDH19	protocadherin 19(PCDH19)	no
PCDH7	protocadherin 7(PCDH7)	no
PCDH9	protocadherin 9(PCDH9)	yes
PCDHA4	protocadherin alpha 4(PCDHA4)	yes
PCDHB1	protocadherin beta 1(PCDHB1)	no
PCDHB15	protocadherin beta 15(PCDHB15)	no
PCDHB2	protocadherin beta 2(PCDHB2)	no
PCDHB5	protocadherin beta 5(PCDHB5)	no
PCDHB7	protocadherin beta 7(PCDHB7)	yes
PCDHGA11	protocadherin gamma subfamily A, 11(PCDHGA11)	yes
PCDHGA7	protocadherin gamma subfamily A, 7(PCDHGA7)	yes
PCDHGB4	protocadherin gamma subfamily B, 4(PCDHGB4)	yes
PCDHGB5	protocadherin gamma subfamily B, 5(PCDHGB5)	yes
PCDHGB7	protocadherin gamma subfamily B, 7(PCDHGB7)	yes
RELN	reelin(RELN)	yes
SPON1	spondin 1(SPON1)	no
SUSD5	sushi domain containing 5(SUSD5)	yes
SVEP1	sushi, von Willebrand factor type A, EGF and pentraxin domain containing 1(SVEP1)	yes and intense
TLN2	talin 2(TLN2)	no
THBS2	thrombospondin 2(THBS2)	no
VCAM1	vascular cell adhesion molecule 1(VCAM1)	yes

Homophilic cell adhesion via plasma membrane adhesion molecules

ID	Gene Name	
MDGA2	MAM domain containing glycosylphosphatidylinositol anchor 2(MDGA2)	yes
CDH11	cadherin 11(CDH11)	yes
CDH13	cadherin 13(CDH13)	yes and intense
CDH2	cadherin 2(CDH2)	yes
CDH6	cadherin 6(CDH6)	yes
CDHR1	cadherin related family member 1(CDHR1)	yes
CLSTN2	calsyntenin 2(CLSTN2)	yes
CADM2	cell adhesion molecule 2(CADM2)	yes and intense
CNTN4	contactin 4(CNTN4)	yes and intense
DCHS1	dachsous cadherin-related 1(DCHS1)	yes
DSG2	desmoglein 2(DSG2)	no
KIRREL3	kirre like nephrin family adhesion molecule 3(KIRREL3)	yes
MUSK	muscle associated receptor tyrosine kinase(MUSK)	yes
NCAM1	neural cell adhesion molecule 1(NCAM1)	yes
PTPRD	protein tyrosine phosphatase receptor type D(PTPRD)	yes
PCDH10	protocadherin 10(PCDH10)	yes
PCDH17	protocadherin 17(PCDH17)	yes
PCDH19	protocadherin 19(PCDH19)	no
PCDH7	protocadherin 7(PCDH7)	no
PCDH9	protocadherin 9(PCDH9)	yes
PCDHA4	protocadherin alpha 4(PCDHA4)	yes
PCDHB1	protocadherin beta 1(PCDHB1)	yes
PCDHB15	protocadherin beta 15(PCDHB15)	no
PCDHB2	protocadherin beta 2(PCDHB2)	no
PCDHB5	protocadherin beta 5(PCDHB5)	no
PCDHB7	protocadherin beta 7(PCDHB7)	yes
PCDHGA11	protocadherin gamma subfamily A, 11(PCDHGA11)	yes
PCDHGA7	protocadherin gamma subfamily A, 7(PCDHGA7)	yes
PCDHGB4	protocadherin gamma subfamily B, 4(PCDHGB4)	yes
PCDHGB5	protocadherin gamma subfamily B, 5(PCDHGB5)	yes
PCDHGB7	protocadherin gamma subfamily B, 7(PCDHGB7)	yes
PRTG	protogenin(PRTG)	yes
SDK1	sidekick cell adhesion molecule 1(SDK1)	yes
TRO	trophinin(TRO)	yes
UNC5C	unc-5 netrin receptor C(UNC5C)	yes

Table S4. Confirmation of genes with the broad H3K4me domain.

Unique to	Gene ID	Track confirmation	Unique to	Gene ID	Track confirmation	Unique to	Gene ID	Track confirmation	Unique to	Gene ID	Track confirmation	Unique to	Gene ID	Track confirmation
CCD	FENDRR	yes	HCT116	TTC31	no	HT29	PYCR1	no	RKO	STKLD1	no	CRC	PIDD1	no
	MEIS1	yes		MAN1B1	no		HOXA3	no		LCN15	no		HGS	no
	AC110813.	yes		MAPK12	no		TRPT1	no		MROH5	no		AC120057.	no
	RND3	yes		AC253536.	no		HELZ2	no		AC138028.	no		PLCB3	no
	FILIP1L	yes		AL807752.	no		AL354836.	no		ARHGEF16	no		RNF40	no
	DUSP10	yes , not in Sig Colon		PHPT1	no		NOL4L	no		NRBP2	no		UCKL1-AS1	no
	PDGFRA	yes		OCEL1	no		KLF5	no		OPLAH	no		CDK4	no
	ZEB2	yes		CIRBP	no		DGAT1	no		PTP4A3	no		DCXR	no
	NKX2-3	yes		TSEN54	no		WDR90	no		AC093525.	no		SYVN1	no
	SGIP1	yes		MIF4GD	no		U2	no		AL589702.	no		MIB2	no

Table S5. Confirmation of genes with H3K4me peaks.

Unique to	Gene ID	Track validation	Unique to	Gene ID	Track validation
Sig colon	TSHZ3	yes	CRC	AC032011.	no
	ADGRL3	yes		SLC5A4-AS	yes
	ISX	yes in SC, not CCD		PAX8-AS1	no
	HMGCS2	yes in SC, not CCD		AC116667.	no
	NCAM1	yes		ECHS1	no
	EPB41L3	yes		NISCH	no
	SDK2	yes in SC, not CCD		RNA5SP18	no
	C11orf96	yes		SLCO1B3	yes
	OCA2	yes		ELFN1-AS1	yes
	TLN2	no		DBP	no

Top 5% Broad with TSS

Unique to	Gene ID	Track validation	Unique to	Gene ID	Track validation	Unique to	Gene ID	Track validation		Unique to	Gene ID	Track validation	Common	Gene ID	Track validation
CCD	MIR99B	no	HCT 116	MIR3190	no	HT-29	BHLHE41	no		RKO	KIF26B	no	to CRC	UBTF	no
	QKI	no		ACBD5	no		SHH	no	in HT-29 and Sig colon		AC025419.	yes		COQ8B	no
	SEMA6A	no		FAT1	no		PRR15	no			MAST4	no		MAML3	no
	GDNF	yes		ESRP1	no		IGF2BP2	no			HIST2H2BC	no		TRIM8	no
	CDC42EP3	no		MIR6805	no		MIR200B	no			MTAP	no		HNRNPF	no
	PCDH18	yes		RRM2	no		AC083923.	no			RNF168	no		MYC	no
	FAM155A	yes		MYADM	no		BRI3	no			SMAGP	yes		KMT2E	no
	ADAMTS5	yes		GPBP1L1	no		ZFP36L2	no			C2CD2	yes		YWHAZ	no
	ATP2A2	yes		TFAP2A	no		PYGB	no			HOXB-AS1	no		CPEB2	no
	CNN3	yes		SPRY4	no		EFNB1	no			GFI1	no		FOXO1	no

Note: Homo sapiens uncharacterized LOC100507065 (LOC100507065), transcript variant 4, long non-coding RNA. (from RefSeq NR_120434)
